# Supplementary material for: Two-dimensional mineral hydrogel-derived single atoms-anchored heterostructures for ultrastable hydrogen evolution
Source: Nat Commun. 2022 Oct 21;13:6249. doi: 10.1038/s41467-022-33725-8 (PMC9586971; doi:10.1038/s41467-022-33725-8)
Supplement: Supplementary file 1 — Supplementary information [file 41467_2022_33725_MOESM1_ESM.pdf]

Supplementary Materials for

Two-dimensional mineral hydrogel-derived single atoms-anchored heterostructures for ultrastable hydrogen evolution

Fucong Lyu[1,2], Shanshan Zeng[3], Zhe Jia[2,4], Fei-Xiang Ma[1,2], Ligang Sun[5,6,*], Lizi Cheng[1,2], Jie Pan[3], Yan Bao[1,2], Zhengyi Mao[1,6], Yu Bu[1,2], Yang Yang Li[3,*], and Jian Lu[1,2,6,7,*]

[1] *Centre for Advanced Structural Materials, City University of Hong Kong Shenzhen Research Institute, Greater Bay Joint Division, Shenyang National Laboratory for Materials Science, Shenzhen 518057, China*

[2] *Hong Kong Branch of National Precious Metals Material Engineering Research Centre, City University of Hong Kong, 83 Tat Chee Avenue, Kowloon, Hong Kong, China*

[3] *Department of Material Science and Engineering, City University of Hong Kong, 83 Tat Chee Avenue, Kowloon, Hong Kong, China*

[4] *School of Materials Science and Engineering, Southeast University, Nanjing 211189, China*

[5] *School of Science, Harbin Institute of Technology, Shenzhen 518055, China*

[6] *Department of Mechanical Engineering, City University of Hong Kong, 83 Tat Chee Avenue, Kowloon, Hong Kong, China*

[7] *CityU-Shenzhen Futian Research Institute, Shenzhen 518045, China*

This PDF file includes:

Supplementary Fig. 1 to 30
Supplementary Table 1 to 14
Supplementary Text
References

Contents

Supplementary Figures

Supplementary Fig. 1 The centrifugation approach to the formation of the mineral hydrogel. 6
Supplementary Fig. 2 Photographs of an amplified 50 times synthetical experiment of the mineral hydrogel. 6
Supplementary Fig. 3 As-obtained FePMoGs with various Fe^{3+}:PMo molar ratios (denoted as ‘x’, which indicates x mol Fe^{3+}:1 mol PMo). 6
Supplementary Fig. 4 Digital images of the concentration-dependent experiment of the FePMo composites with various molar ratios of Fe^{3+} to PMo from left to right: 6:1, 12:1, 19:1, 25:1, 31:1, and 37:1 (the indexed time was the estimated time for fully self-assembly process). 7
Supplementary Fig. 5 Concentration-dependent experiment SEM images of the FePMo composites obtained after self-assembly process with various molar ratios of Fe^{3+} to PMo: 6:1 (a), 12:1 (b), 19:1 (c), 25:1 (d), 31:1 (e), and 37:1 (f). 7
Supplementary Fig. 6 Digital images of the time-dependent experiment of the FePMo nanosheets. The molar ratio of Fe^{3+} to PMo reagents is 25:1. 12
Supplementary Fig. 7 SEM images of the time-dependent evolution of wrinkle-like FePMoG: 1h (a), 2 h (b), 4 h (c), 8 h (d), 16 h (e) and 24 h (f). The molar ratio of Fe^{3+} to PMo reagents is 25:1; (g) schematic illustration of the FePMoG assemble process. 13
Supplementary Fig. 8 Optical photograph of the mineral hydrogels contaning added ion species (~ 3 at.% of the sum of Fe and Mo atoms, the molar ratio of Fe^{3+}/PMo is 25:1). 14
Supplementary Fig. 9 (a) XRD patterns, (b) FTIR spectra of the FePMoG with the molar ratio of Fe^{3+} to PMo reagents is 25:1. 14
Supplementary Fig. 10 Mo 3*d* spectrum of FePMoG. 15
Supplementary Fig. 11 XRD patterns of samples obtained at different phosphorization temperatures. 15
Supplementary Fig. 12 (a) Low magnification TEM image, (b) HRTEM image of bulk FeMoP-500. 16
Supplementary Fig. 13 (a) Low magnification TEM image, (b) High magnification bright-field TEM image, (c) Dark-field TEM image, (d) high-resolution TEM image, (e) SEAD pattern, (f) STEM image and the corresponding EDS elemental mapping of Fe (green), Mo (violet), P (orange) and O (cyan) of the FeMoP-450. 17
Supplementary Fig. 14 (a) Low magnification TEM image, (b) High magnification bright-field TEM image, (c) Dark-field TEM image, (d) high-resolution TEM image, (e) SEAD pattern, (f) STEM image and the corresponding EDS elemental mapping of Fe (green), Mo (violet), P (orange) and O (cyan) of the FeMoP-550. 18

Supplementary Fig. 15 (a) Nitrogen sorption isotherms, (b) pore size distributions of the FeMoP-450, Fe/SAs@Mo-based-HNSs and FeMoP-550 catalysts. 19
Supplementary Fig. 16 XPS spectrum of Fe/SAs@Mo-based-HNSs. 19
Supplementary Fig. 17 (a) The corresponding k^2-weighted FT-EXAFS spectra and fitting line of Fe/SAs@Mo-based-HNSs at Mo K-edge in the R spacing, Wavelet transforms for the k^2-weighted EXAFS signals at Mo *K*-edge of (b) Mo foil, (c) MoO_2, and (d) MoO_3. 20
Supplementary Fig. 18 (a) The corresponding k^3-weighted FT-EXAFS spectra and fitting line of Fe/SAs@Mo-based-HNSs at Fe K-edge in the R spacing, Wavelet transforms for the k^3-weighted EXAFS signals at Fe *K*-edge of (b) Fe foil, (c) Fe_3O_4, and (d) Fe_2O_3. 21
Supplementary Fig. 19 O 1*s* XPS spectra of Fe/SAs@Mo-based-HNSs. 22
Supplementary Fig. 20 Spectroscopy of FeMoP-550 at Fe K-edge: (a) Fe K-edge XANES spectra; (b) corresponding k^3-weighted FT of EXAFS spectra; (c) the corresponding k^3-weighted FT-EXAFS spectra and fitting line in the R spacing; and (d) wavelet transforms for k^3-weighted EXAFS signals. 24
Supplementary Fig. 21 (a) Polarization curves of the FeMoP samples in 1 M KOH with iR correction. (b) Corresponding Tafel plots. 25
Supplementary Fig. 22 Polarization curves of three independent Fe/SAs@Mo-based-HNSs prepared from different batches in 1 M KOH with iR correction. 25
Supplementary Fig. 23 Cyclic voltammograms in the region of 0-0.1 V vs. RHE at various scan rates for (a) FeMoP-450, (b) Fe/SAs@Mo-based-HNSs, (c) FeMoP-550, and (d) Bulk FeMoP-500...26
Supplementary Fig. 24 Polarisation curves of Fe/SAs@Mo-based-HNSs before and after 500 h test at a scan rate of 5 mV s^{-1} with potential error (iR) correction 27
Supplementary Fig. 25 (a) Low magnification TEM image, (b) High magnification TEM image, (c) high-resolution TEM image, (d) STEM image and the corresponding EDS elemental mapping of Fe (green), Mo (violet), P (orange) and O (cyan) of the Fe/SAs@Mo-based-HNSs after 500 h test. 27
Supplementary Fig. 26 XRD pattern of the Fe/SAs@Mo-based-HNSs after 500 h test (blue curve). The peaks indexed with mark * derived from the carbon paper as the sample load on the carbon paper. 28
Supplementary Fig. 27 (a) Mo 3*d*, (b) Fe 2*p* spectrum, (c) P 2*p* spectrum, and (d) O 1*s* spectrum of the Fe/SAs@Mo-based-HNSs after 500 h test. 28
Supplementary Fig. 28 Partial density of states (PDOS) of MoP, MoP_2, MoO_2, MoP/MoP_2, MoP/MoO_2, MoP_2/MoO_2, Fe@MoO_2-1 and Fe@MoO_2-2 after H_2O adsorption at surface sites. The black dashed lines at the energy of zero indicate the Fermi level (E_F). 32

Supplementary Fig. 29 Free energy diagrams of reaction coordinate for water dissociation on the surfaces of single-phase models (MoP, MoP_2 and MoO_2), heterostructured interface models (MoP/MoP_2, MoP/MoO_2 and MoP_2/MoO_2), monoatomic dispersed Fe onto MoO_2 surface models (Fe@MoO_2-1 and Fe@MoO_2-2) and Pt(111). 33

Supplementary Fig. 30 The representative atomic configurations after H adsorption at the surface sites of MoP, MoP_2 and MoO_2 with corresponding ΔG_{H^*}. 34

Supplementary Fig. 31 DFT results of 2D electron density differences after adsorption of H* onto the active sites in the single-phase models. Red and blue represent the depletion and accumulation of electrons with the unit of e/Å^3, respectively. 34

Supplementary Fig. 32 The *d*-orbital partial density of states (*d*-PDOS) of three single-phase and two heterostructured interface models with H adsorption onto Mo top. The solid lines marked with digitals show the position of d-band centers and the dashed line indicate Fermi level (E_F). The corresponding ΔG_{H^*} values for the models are also indicated. 35

Supplementary Tables

Supplementary Table 1 Comparison of mineral hydrogel, porous framework and carbon substrate used for single atom-based catalyst production 8

Supplementary Table 2 Chemical compositions of samples obtained at different phosphorization temperatures determined by XRD... 16

Supplementary Table 3 EXAFS fitting parameters at the Mo K-edge for various samples（S_0^2=0.94） 20

Supplementary Table 4 EXAFS fitting parameters at the Fe K-edge for various samples（S_0^2=0.94） 21

Supplementary Table 5 Elemental compositions for FePMoG, FeMoP-450, Fe/SAs@Mo-based-HNSs, FeMoP-550 and tested FeMoP-500 determined by XPS. 22

Supplementary Table 6 Elemental compositions for FePMoG, FeMoP-450, Fe/SAs@Mo-based-HNSs and FeMoP-550 determined by TEM EDS... 23

Supplementary Table 7 Elemental compositions for FePMoG, FeMoP-450, Fe/SAs@Mo-based-HNSs and FeMoP-550 determined by ICP-OES. 23

Supplementary Table 8 EXAFS fitting parameters at the Fe K-edge for various samples（S_0^2=0.74） 24

Supplementary Table 9 Comparison of HER performance in 1 M KOH for FeMoP-T catalysts. .. 25

Supplementary Table 10 Amount of hydrogen production for samples at a constant current density of 10 mA/cm^2.* 26

Supplementary Table 11 ICP-OES results of the Fe/SAs@Mo-based-HNSs after stability test for 500h at 20 mA cm^{-2} current density. 28
Supplementary Table 12 Comparison of HER performance in 1 M KOH for Fe/SAs@Mo-based-HNSs and previous reported catalysts..29
Supplementary Table 13 The H_2O adsorption energy, adsorption site and the bonding distance between active site and O atom in H_2O..31
Supplementary Table 14 The ΔG_{H*} and *d*-band centers of three single-phase and two heterostructured interface models with H adsorption onto Mo top. 36

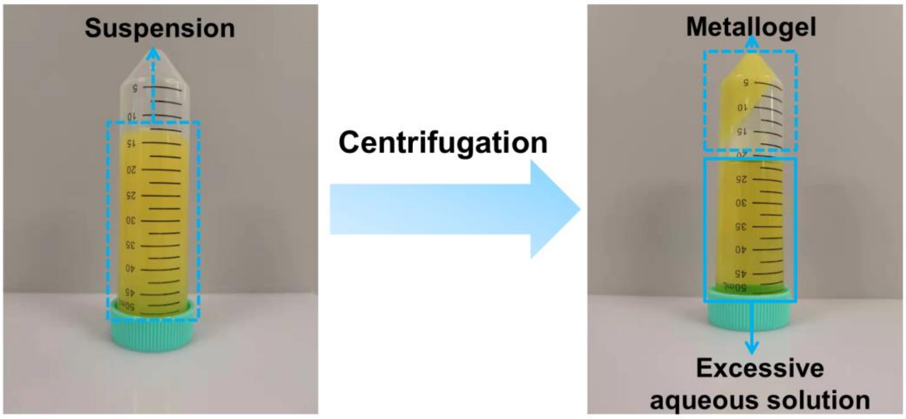

Supplementary Fig. 1 The centrifugation approach to the formation of the mineral hydrogel.

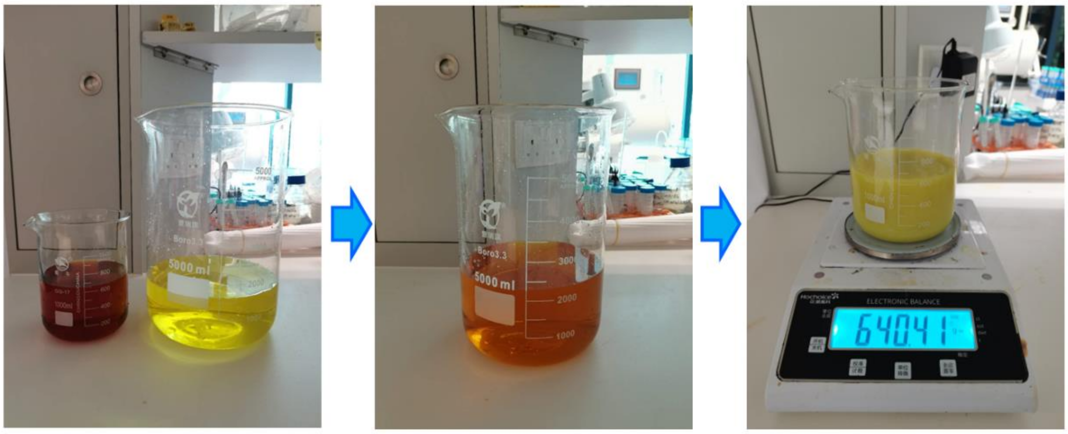

Supplementary Fig. 2 Photographs of an amplified 50 times synthetical experiment of the mineral hydrogel.

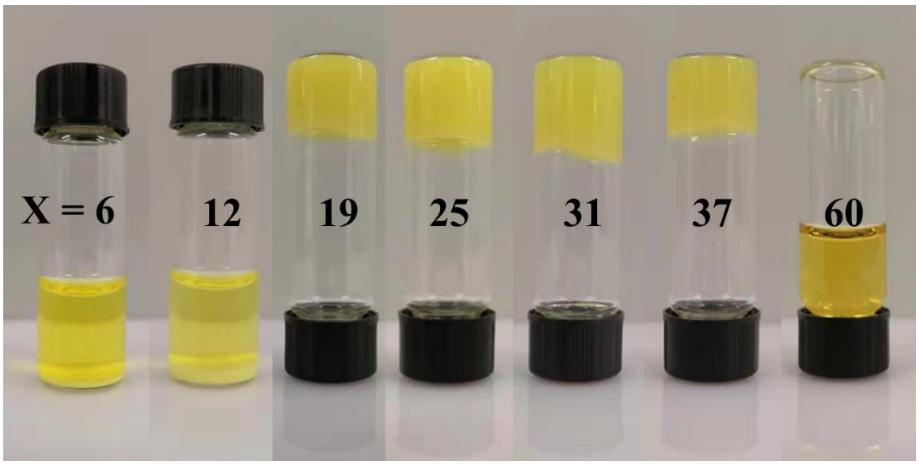

Supplementary Fig. 3 As-obtained FePMoGs with various Fe^{3+}:PMo molar ratios (denoted as 'x', which indicates x mol Fe^{3+}:1 mol PMo).

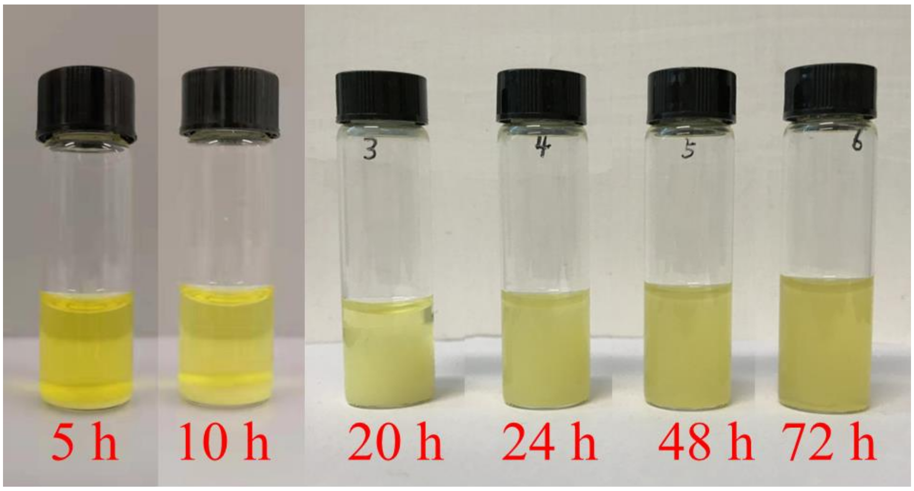

Supplementary Fig. 4 Digital images of the concentration-dependent experiment of the FePMo composites with various molar ratios of Fe^{3+} to PMo from left to right: 6:1, 12:1, 19:1, 25:1, 31:1, and 37:1 (the indexed time was the estimated time for fully self-assembly process).

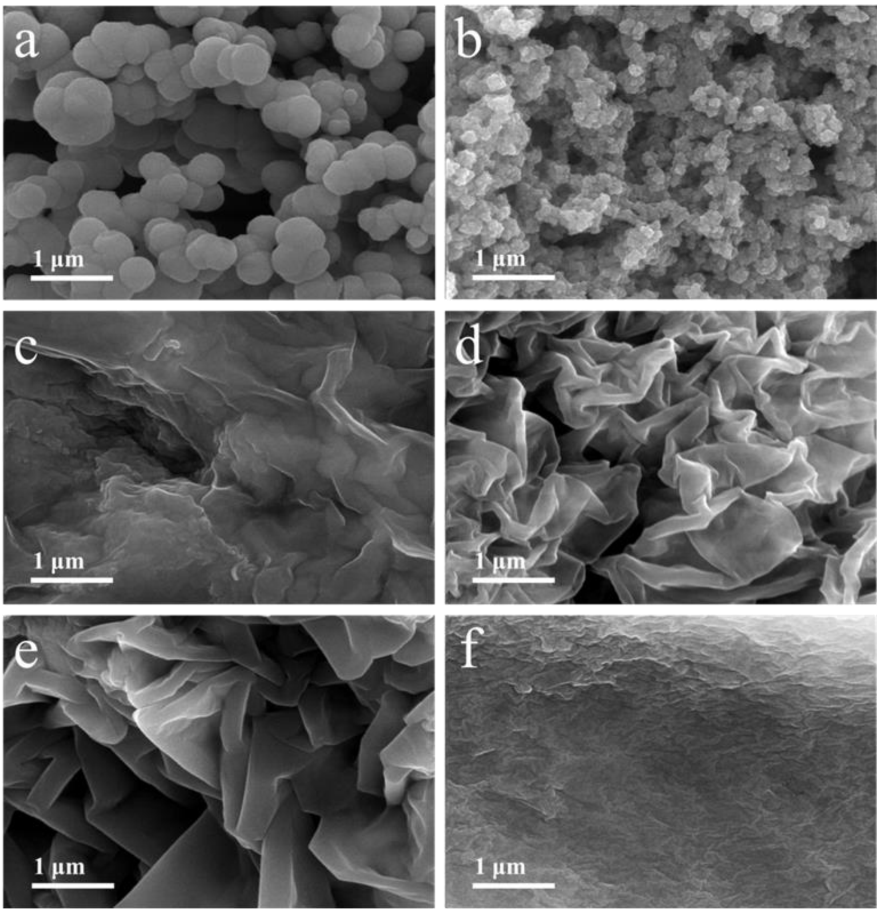

Supplementary Fig. 5 Concentration-dependent experiment SEM images of the FePMo composites obtained after self-assembly process with various molar ratios of Fe^{3+} to PMo: 6:1 (a), 12:1 (b), 19:1 (c), 25:1 (d), 31:1 (e), and 37:1 (f).

A concentration-dependent experiment was carried out with various molar ratios of Fe^{3+}:

PMo (6:1, 12:1, 19:1, 25:1, 31:1, 37:1, and 60:1), and it can be clearly seen that some of these mixtures precipitated faster than others (Supplementary Fig. 3, 4). Specifically, precipitates of FePMoG were formed within minutes in lower molar ratio (6:1 and 12:1) mixtures of Fe^{3+}: PMo and within several hours in higher molar ratio (19:1, 25:1, 31:1, and 37:1) mixtures of Fe^{3+}: PMo; however, no precipitate was formed in the highest molar ratio (60:1) mixture of Fe^{3+}: PMo.

As shown in Supplementary Fig. 4, 5a, when the ratio Fe^{3+}/PMo is as low as 6:1 forms a sphere-like shape with a diameter of about several hundred nanometers that is comprised of several spheres grown together. When the ratio is increased to 12:1, its morphology consists of uniform nanoparticles that are significantly smaller than that of the 6:1 (Supplementary Fig. 5b). When the ratio is increased again to 19:1, the morphology is totally different, as depicted in Supplementary Fig. 5c, it seems to form a nanosheet-like cluster, but it is like a rudiment of the whole nanosheet, and in some places, it appears to consist of many agminated nanoparticles covered by a thin film. It's necessary to note that partially deposited on the bottom of the bottle and the upper liquid near to the solution surface became transparent. At a ratio of 25:1, Fe^{3+} and PMo obviously self-assembled into wrinkle-like nanosheets (Supplementary Fig. 5d). At a ratio of 31:1, they self-assembled into a wrinkle nanosheet (Supplementary Fig. 5e) with a significantly higher thickness than that of 25:1. At a ratio of 37:1, it took much longer to form precipitates, and the nanosheet was very large with vast wrinkles at the surface (Supplementary Fig. 5f). Too high ratio led to overly lengthy assembly (when the molar ratio of Fe^{3+}/PMo was 50:1, not shown here, self-assembly took about seven days), and when the molar ratio of Fe^{3+}/PMo was as high as 60:1, the solution kept transparent. It can be concluded from this concentration-dependent experiment that the morphology of the FePMo composites is significantly dependent on the ratio of Fe^{3+} to POM, and the higher ratios of Fe^{3+}/PMo were associated with a longer self-assembly process.

Supplementary Table 1 Comparison of mineral hydrogel, porous framework and carbon substrate used for single atom-based catalyst production

		**Mineral hydrogel**		**Porous framework**		**Carbon**	
	content	details	Score[a]	details (e.g. Ref[1])	Score[a]	details (e.g. Ref[2])	Score[a]
**facile fabrication**	instrument	centrifuge	10	oven, ice machine, heating agitator, glass reactor, freezer dryer, vacuum pump, filter, sonicator, centrifuge, tube furnace	2	glass beaker, heating agitator, oven, tube furnace, centrifuge, sonicator	3
	procedure	standing, centrifugation,	10	stirring, cooling, heating, vacuum filtration, freeze dry, conjugation, polymerization,	2	stirring, drying, heating, noble metal deposition, sonication, heating and stirring,	3

				sonication		centrifugation, drying	
	template	no need	10	no need	10	need release gas to assist the formation of nanosheet	5
	purification	water wash	10	washed with degassed ethanol and diethyl ether, solvothermal wash with organic solvent	3	water and ethanol wash	5
	reaction condition	room temperature	10	ice bath, heated at 120 °C，heated at 300 °C	2	drying at 80 °C，heated at 900 °C, sonication, stirring at 80 °C	2
	total access	simple synthetic procedure, equipment	100	complex synthetic procedure, reaction conditions are complex and numerous	40	complex synthetic procedure, reaction conditions are complex and numerous	30
**green synthesis**	Materials	$Fe(NO_3)_3·9H_2O$, phosphomolybdic acid, NaCl, NaH_2PO_2, ethanol, deionized water	10	$CoCl_2·6H_2O$, chloranilic acid, H_2SO_4, ethylenediamine, 1-methy-2-pyrolidinone (NMP), anhydrous NMP, diethyl ether, ethanol, deionized water	1	urea, glucose, $Ni(CH_3COO)_2$ • $4H_2O$, ethanol, ethylene glycol, H_2PtCl_6, ethanol, deionized water, ammonium hydroxide	8
	yield (from raw material to final catalyst)	~80%	8	Synthesis of hexaaminobenzene: ~39.69% conjugation: 50.89% polymerization: 90%	1.5	carbonization: usually <30%	3
	organic release (except ethanol)	none	10	isopropanol, NMP, anhydrous NMP, ethylenediamine, diethyl ether is easy to evaporate	1	Organic waste during the high temperature pyrolysis	1
	solvent	deionized water	10	isopropanol, NMP, ethylenediamine, diethyl ether	1	ethylene glycol and ethanol mixture	1
	Catalyst usage	no	10	Some frame works preparation need catalyst, e.g. covalent organic framework[3]	3	CVD method to synthesize the carbon need grow the catalyst beforehand[4]	4
	recycle	few kinds of	10	Some organic	5	the kind of	

		ions in liquid waste, easy to disposal		solvent can recycle but it is complicate to separate		organic solvent is less and easy to recycle,	
	total access	using common non-toxic inorganic salt, easy recycle	100	using multiple organic solution and some are toxic	10	using common non-toxic inorganic salt, easy recycle	50
**time efficiency**	precursor synthesis	20 h	8	64 h	3	18 h	8.5
	subsequent synthesis	16.5 h	9	81 h	1	34 h	3
	total access	no equipment needed, just few more cans can produce mineral hydrogel simultaneously	100	some MOF even need several week to prepare and purification[5]	20	if use MOF and COF as carbon source, the synthetic time will increase greatly	40
**morphology controllability**	1D	Ref[6]	10	Ref[7]	6	Ref[8,9]	3
	2D	This work	10	Ref[1,10]	5	Ref[2,11]	6
	3D	Ref[12-14]	10	Ref[15]	9	Ref[16]	9
	total access	easy to control the morphology	100	need to change a lot of reagents, reaction condition, even develop a new method	60	need to change a lot of reagents, reaction condition, even develop a new method, involving using the catalyst and using the porous framework to prepare	35
**Organic free**	raw material	yes	10	no	0	no, but using common organic solvent with low toxicity	5
	products	yes	10	the intermediate products are organic	1	organic waste produced during the high temperature pyrolysis	5
	total access	no organic species during the whole preparation process of mineral hydrogel	100	using a variety of and large amount organic solvents and some are toxic and complicate to prepare	5	the glucose and ethylene glycol is abundant and non-toxic	50
**abundance**	source	resource of inorganic salts is rich and easy to process	10	the synthesis of some organic solvent is complex and their yield is low	1	noble metal salt is rare and expensive; the organic solvent used in easy to produce	3
	total access	rich in raw materials and easy to prepare	100	using a variety of and large amount organic solvents	5	all regent except the noble metal salt is rich and	60

				and some are toxic and complicate to prepare		common; many carbon-based catalyst didn't need to use the noble metal	
**universality**	ion species	majority metal ion can be added in the mineral hydrogel	9	Ref[1] can use multiple metal ion for conjugation. However, majority need special metal that can coordinate with linker, or absorb limited metal ion[17-20]	4	many metal ions can be absorbed in the carbon or carbon precursor, the use of noble metal limit the universality	6
	synthetic method	simple, directly add other metal ion in the precursor solution	10	need to consider the coordinated property of the metal ions, or absorb ability of the framework	3	Need to consider the property of metal ion; the further deposition of noble metal is low efficiency	3
	total access	easy to add other metal ion and have large potential in prepare other metal single atom catalyst	100	can prepare other single atom catalyst but the efficiency and yield is low, synthetic method is complex	30	can prepare other single atom catalyst but the efficiency and yield is low, synthetic method is complex	60
**Low cost** (USD, per kg produced catalyst)	materials cost	35.39	10	4779.64	2	8045.85	1
	electricity consumption	0.49	10	97.44	1	47.78	4
	total access	only using abundant inorganic salts; simple preparation process; few instruments are used	100	need use many special organic solvent; rare in resources; complex production process; use many different instrument	10	the noble metal salt and some raw material is expensive; not rich in resources; need to use large amount of organic solvent, multiple preparation steps; some method didn't use noble metal and the cost will reduce a lot[21]	70

[a] These scores are based on the practical value, or complication degree, toxicity (in the range of 0-10); total access score of mineral hydrogel is normalized to 100; the details were refer to the example of 2D porous frame work and carbon.

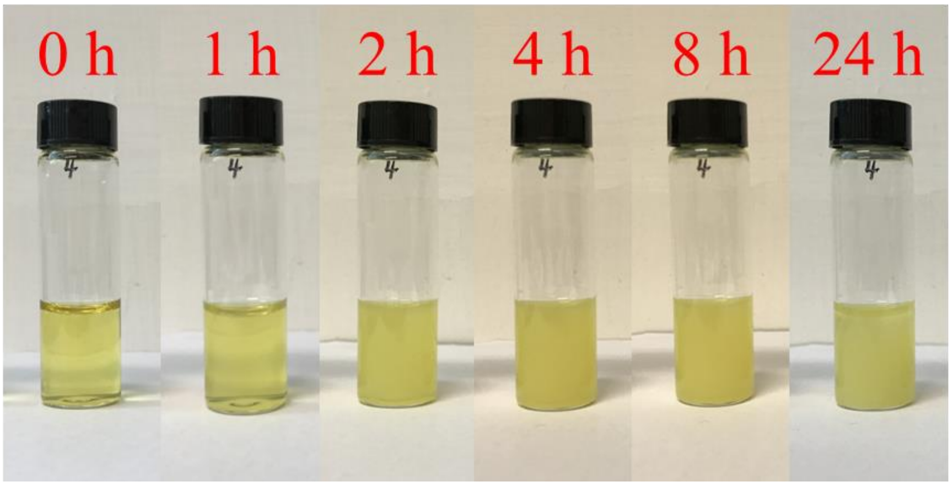

Supplementary Fig. 6 Digital images of the time-dependent experiment of the FePMo nanosheets. The molar ratio of Fe^{3+} to PMo reagents is 25:1.

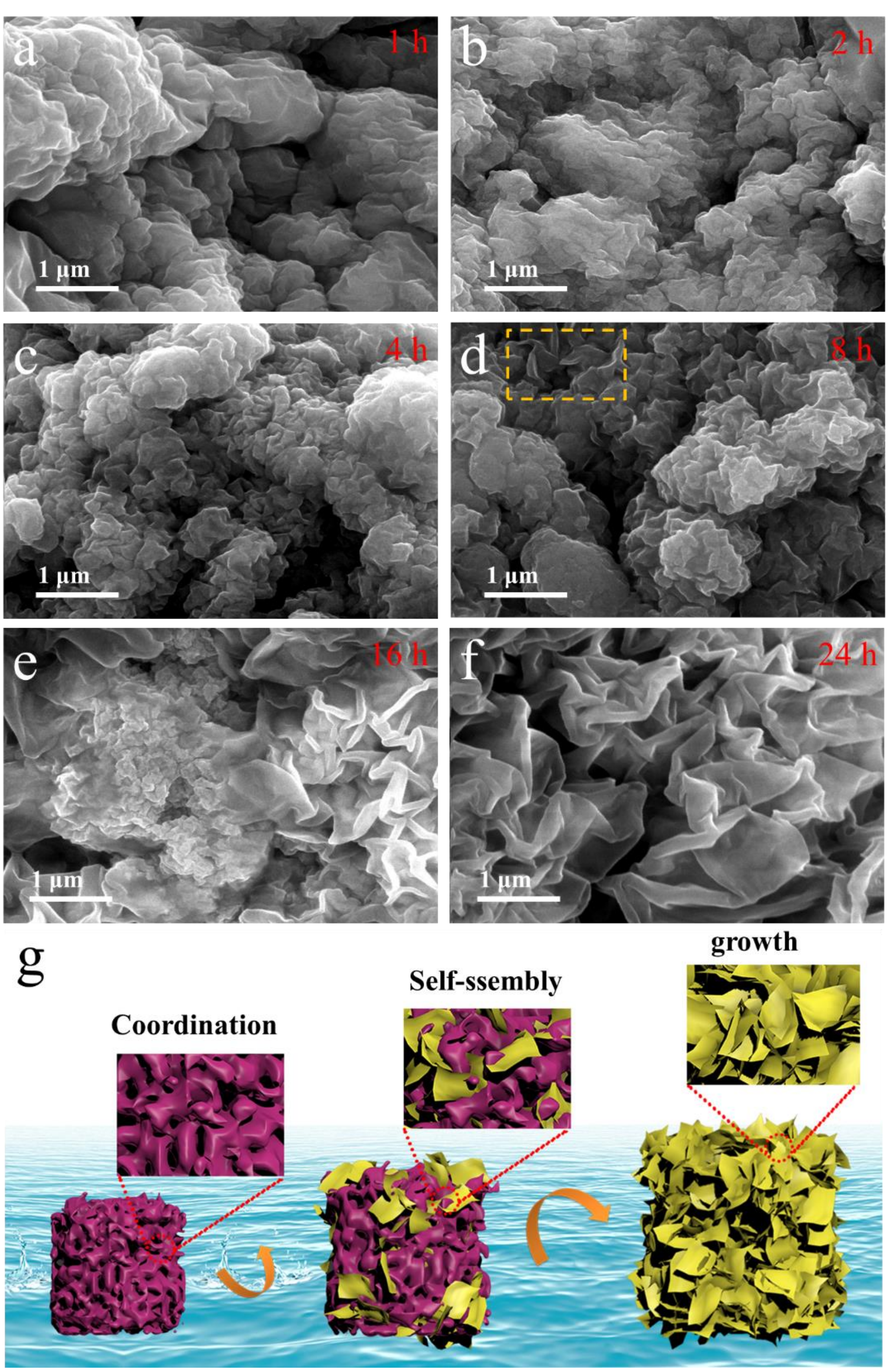

Supplementary Fig. 7 SEM images of the time-dependent evolution of wrinkle-like FePMoG: 1h (a), 2 h (b), 4 h (c), 8 h (d), 16 h (e) and 24 h (f). The molar ratio of Fe^{3+} to PMo reagents is 25:1; (g) schematic illustration of the FePMoG assemble process.

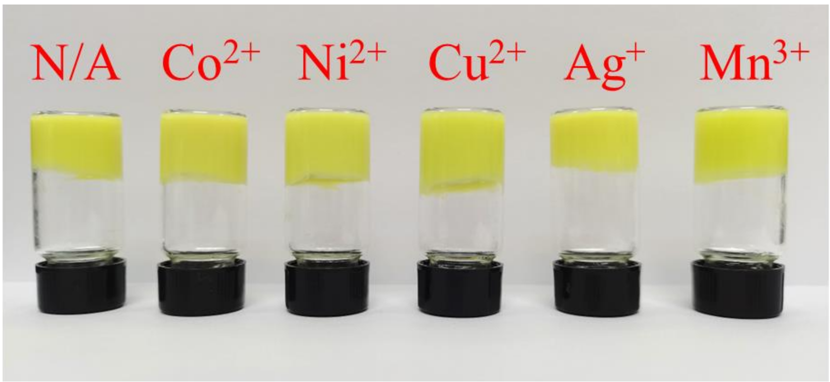

Supplementary Fig. 8 Optical photograph of the mineral hydrogels contaning added ion species (~ 3 at.% of the sum of Fe and Mo atoms, the molar ratio of Fe^{3+}/PMo is 25:1).

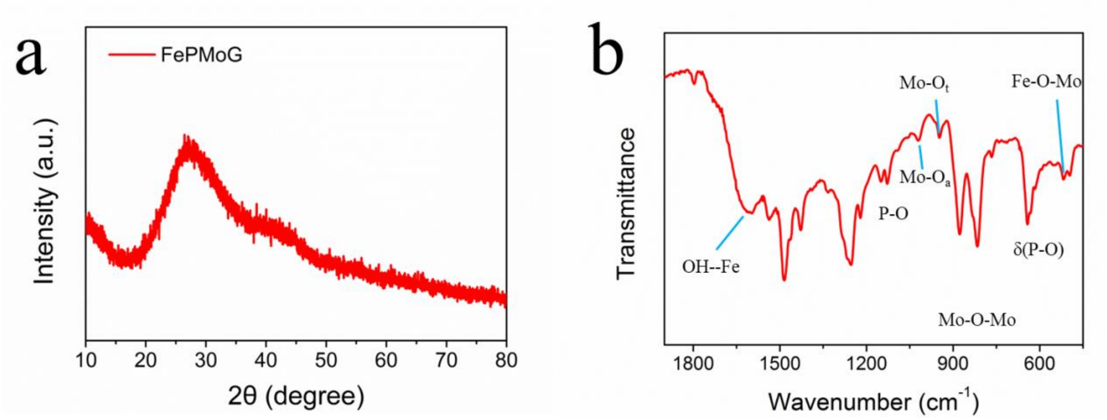

Supplementary Fig. 9 (a) XRD patterns, (b) FTIR spectra of the FePMoG with the molar ratio of Fe^{3+} to PMo reagents is 25:1.

The bands located at 1147 and 1126 cm^{-1} represent asymmetric stretching vibrations (ν_{as}) of P–O_a, and the band centred at 642 cm^{-1} represents a δ (P–O) vibration. The bands at 1017 cm^{-1} (ν_{as} (Mo–O_a)), 945 cm^{-1} (ν_{as} (Mo–O_t)), and the triplet at 876 cm^{-1} (ν_{as} (Mo–O_a–Mo)), 816 cm^{-1} (ν_{as} (Mo–O_c–Mo)), and 760 cm^{-1} (ν_{as} (Mo–O_b–Mo)) represent the interactions between Mo and various forms of O. The red and blue shifts in the vibrations of the Keggin structure result from the interaction between Fe^{3+} and PMo–heteropolyanions [22-24]. In addition, the broad band at 1625 cm^{-1} represents the asymmetric stretching mode of the OH coordinated to Fe^{3+}, and the bands at 515 and 495 cm^{-1} are due to an Fe–O–Mo vibration[22,23].

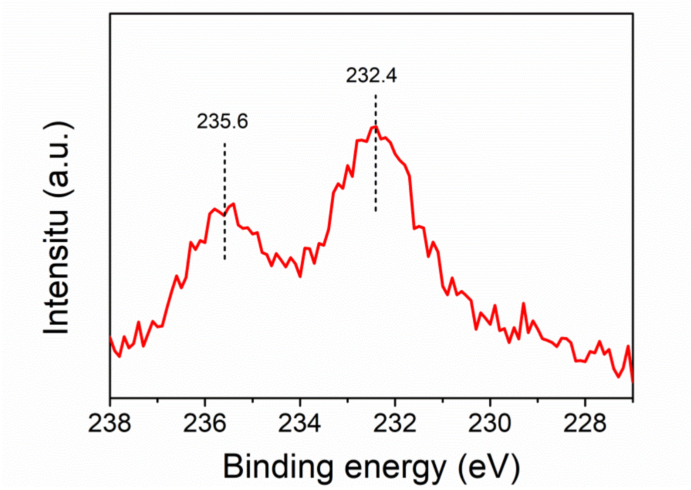

Supplementary Fig. 10 Mo 3*d* spectrum of FePMoG.

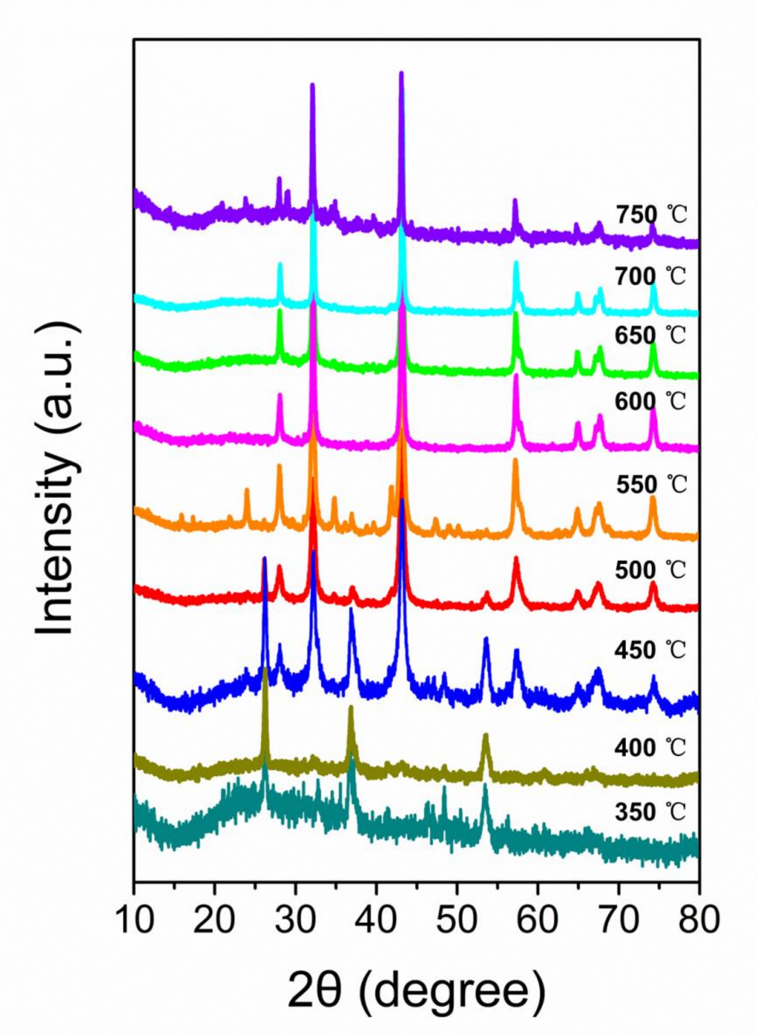

Supplementary Fig. 11 XRD patterns of samples obtained at different phosphorization temperatures.

The main phases at 27.95°, 32.17°, 43.15°, 57.48°, 64.93°, 67.86°, and 74.33° correspond to the (001), (100), (101), (110), (111), (102), and (201) crystal planes of MoP (JCPDS No. 24-0771), respectively. The diffraction peaks at 25.99°, 37.35°, and

53.46° represent the (011), (111), and (022) crystal planes of MoO_2 (JCPDS No. 78-1069), respectively. Other weak diffraction peaks at 23.90°, 34.64°, 41.68°, 47.20°, 48.91°, and 49.99° are ascribable to the (021), (111), (131), (112), (042), and (150) planes of MoP_2 (JCPDS No. 89-2678), respectively.

Supplementary Table 2 Chemical compositions of samples obtained at different phosphorization temperatures determined by XRD.

Temperatures (°C)	FeP	MoO_2	MoP	MoP_2
350	✓	✓	—	—
400	✓	✓	✓	—
450	✓	✓	✓	✓
500	—	✓	✓	✓
550	—	✓	✓	✓
600	—	—	✓	✓
650	—	—	✓	✓
700	—	—	✓	✓
750	—	—	✓	✓

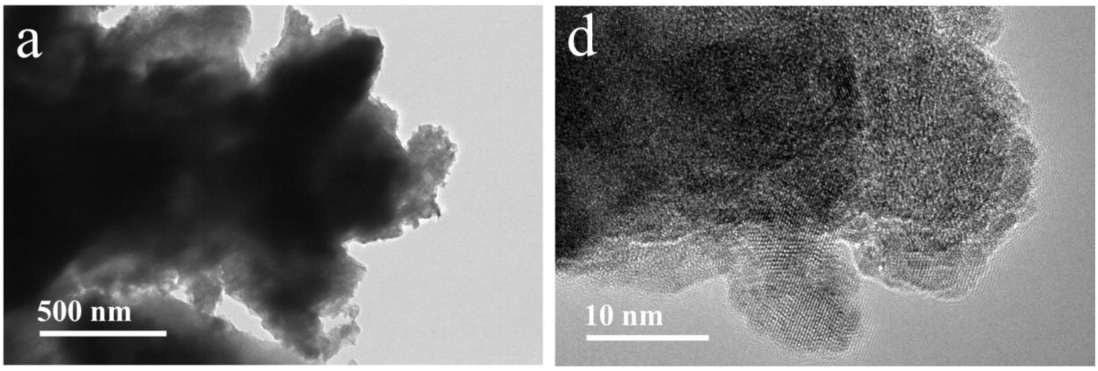

Supplementary Fig. 12 (a) Low magnification TEM image, (b) HRTEM image of bulk FeMoP-500.

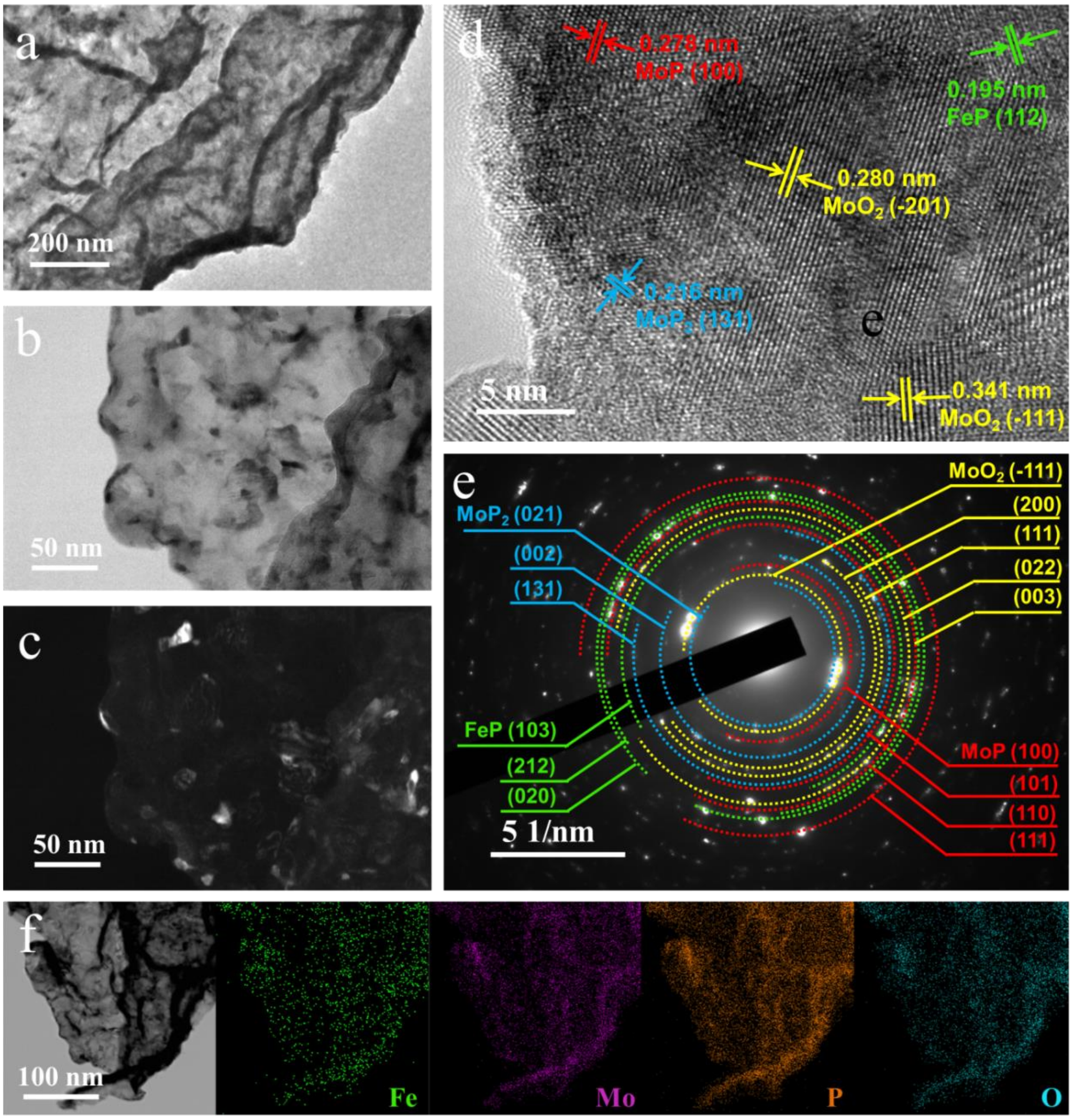

Supplementary Fig. 13 (a) Low magnification TEM image, (b) High magnification bright-field TEM image, (c) Dark-field TEM image, (d) high-resolution TEM image, (e) SEAD pattern, (f) STEM image and the corresponding EDS elemental mapping of Fe (green), Mo (violet), P (orange) and O (cyan) of the FeMoP-450.

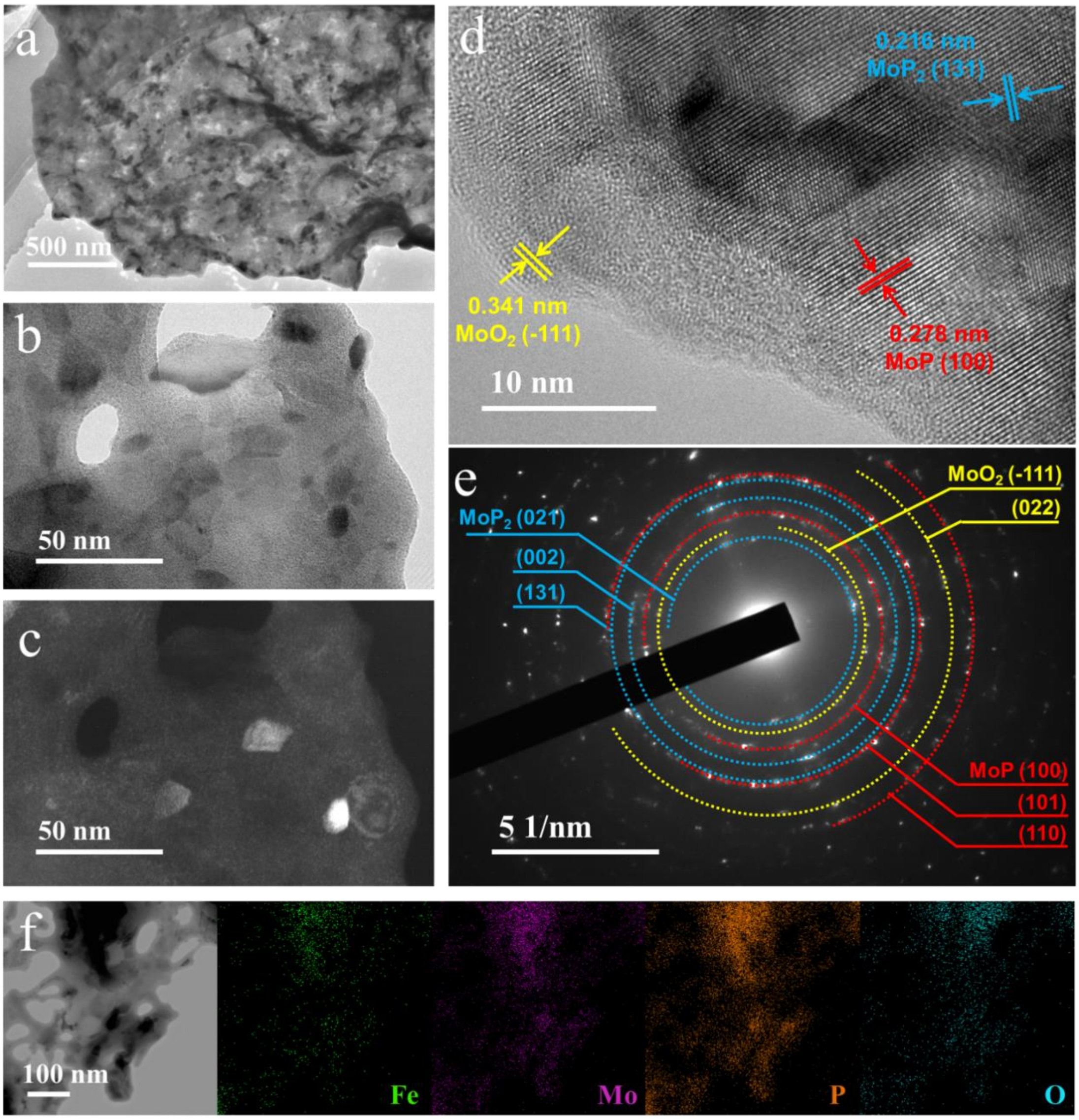

Supplementary Fig. 14 (a) Low magnification TEM image, (b) High magnification bright-field TEM image, (c) Dark-field TEM image, (d) high-resolution TEM image, (e) SEAD pattern, (f) STEM image and the corresponding EDS elemental mapping of Fe (green), Mo (violet), P (orange) and O (cyan) of the FeMoP-550.

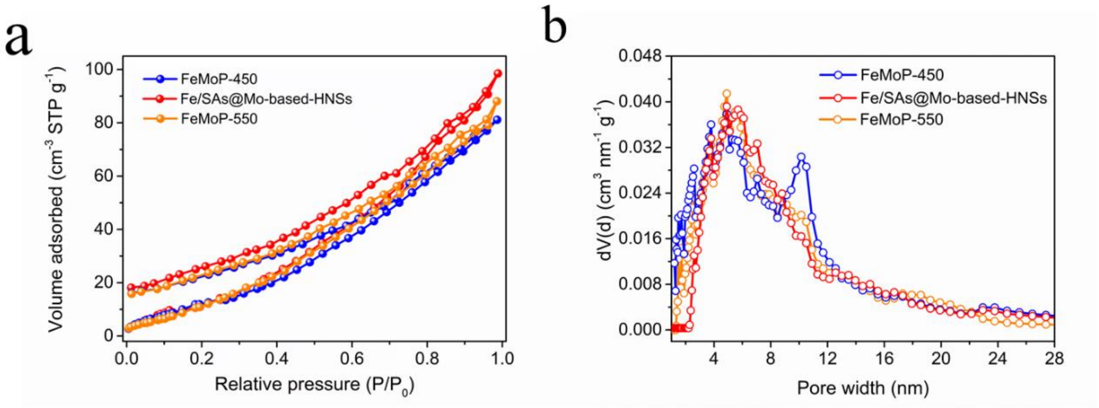

Supplementary Fig. 15 (a) Nitrogen sorption isotherms, (b) pore size distributions of the FeMoP-450, Fe/SAs@Mo-based-HNSs and FeMoP-550 catalysts.

The Brunauer–Emmett–Teller (BET) surface area of the Fe/SAs@Mo-based-HNSs was found to be 89.6 $m^2\ g^{-1}$, which is higher than that of FeMoP-450 and FeMoP-550 (81.7 $m^2\ g^{-1}$ and 86.1 $m^2\ g^{-1}$, respectively) and further demonstrates Fe/SAs@Mo-based-HNSs' intrinsically porous structure (Supplementary Fig. 15). The pore-size distribution of the Fe/SAs@Mo-based-HNSs clearly indicates that they have many mesopores with diameters of 1.3–12 nm, which would provide numerous exposed active sites. The similar BET surface areas and pore-size distributions of FeMoP-450 and FeMoP-550 suggest that the phosphorisation temperature in the heterostructure-forming range has only a minor effect on the formation of a porous structure.

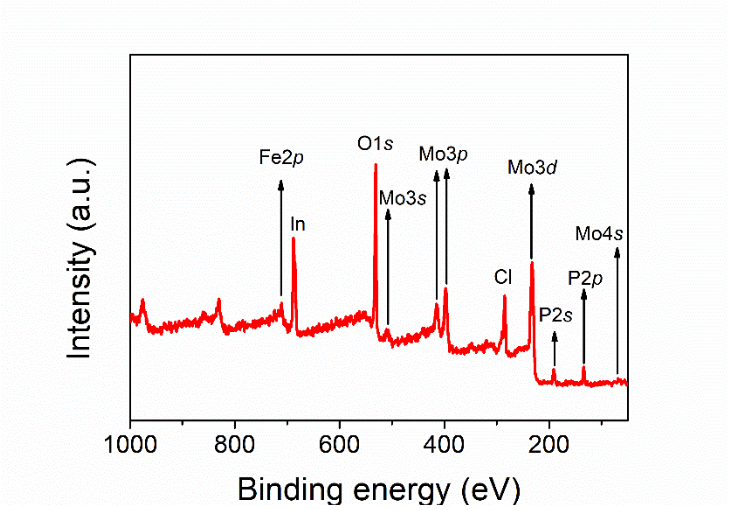

Supplementary Fig. 16 XPS spectrum of Fe/SAs@Mo-based-HNSs.

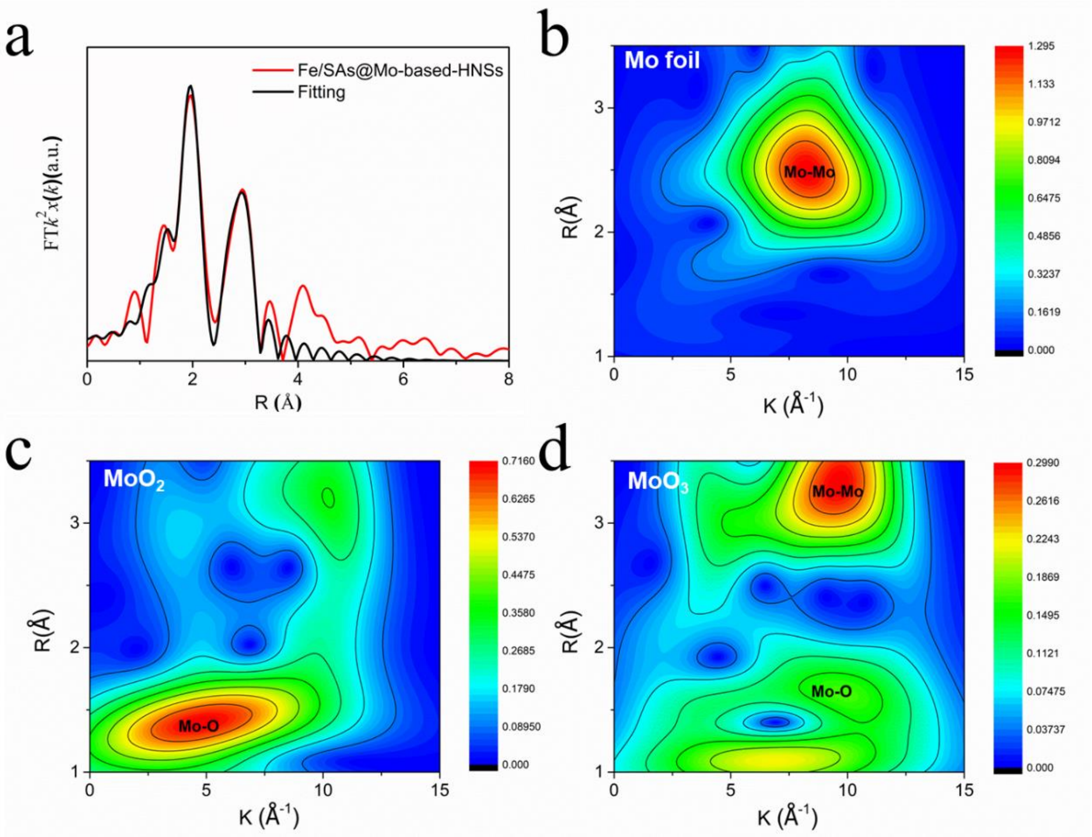

Supplementary Fig. 17 (a) The corresponding k^2-weighted FT-EXAFS spectra and fitting line of Fe/SAs@Mo-based-HNSs at Mo K-edge in the R spacing, Wavelet transforms for the k^2-weighted EXAFS signals at Mo *K*-edge of (b) Mo foil, (c) MoO_2, and (d) MoO_3.

Supplementary Table 3 EXAFS fitting parameters at the Mo K-edge for various samples（S_0^2=0.94）

	shell	CN	R(Å)	σ^2	ΔE_0	R factor
Mo foil	Mo-Mo	8	2.72±0.01	0.0039	4.1±0.6	0.0022
	Mo-Mo	6	3.14±0.01	0.0040		
Fe/SAs@Mo-based HNSs	Mo-O	0.5±0.2	1.99±0.03	0.0033	2.6±1.3	0.0118
	Mo-P	3.0±0.2	2.44±0.01	0.0048		
	Mo-O	2.5±0.2	3.20±0.01	0.0035		

[a]*N*: coordination numbers; [b]*R*: bond distance; [c]σ^2: Debye-Waller factors; [d] ΔE_0: the inner potential correction. *R* factor: goodness of fit.

In the FT-EXAFS spectra and fitting line at Mo K-edge, the peaks at 1.45, 1.95 and 2,93 Å, which could be attributed to Mo-O, Mo-P and Mo-O bonds, respectively. Comparing with Mo foil, no apparent peaks (2.72 and 3.14 A) for Mo-Mo bonds are detected in both Fe/SAs@Mo-based-HNSs, confirming the absence of Mo nanoparticles.

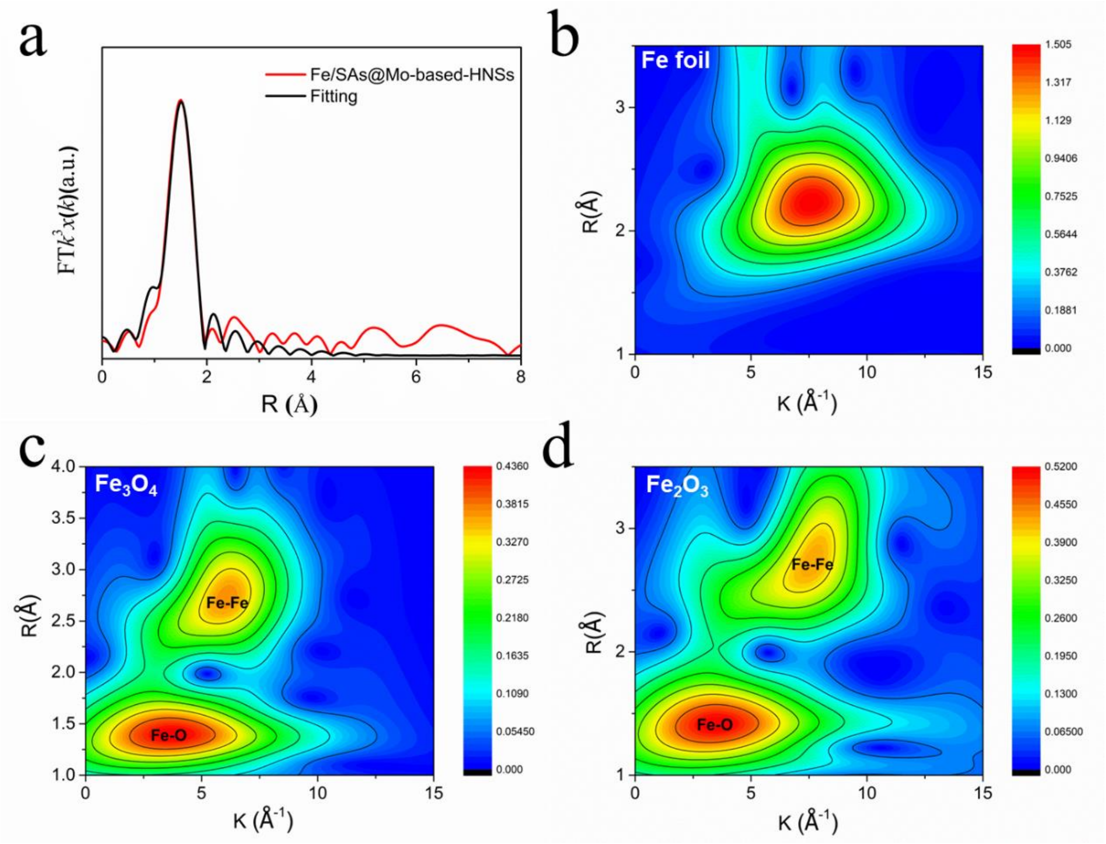

Supplementary Fig. 18 (a) The corresponding k^3-weighted FT-EXAFS spectra and fitting line of Fe/SAs@Mo-based-HNSs at Fe K-edge in the R spacing, Wavelet transforms for the k^3-weighted EXAFS signals at Fe *K*-edge of (b) Fe foil, (c) Fe_3O_4, and (d) Fe_2O_3.

Supplementary Table 4 EXAFS fitting parameters at the Fe K-edge for various samples（S_0^2=0.94）

	shell	CN	R(Å)	σ^2	ΔE_0	R factor
Fe foil	Fe-Fe	8	2.47±0.01	0.0052	7.1±1.1	0.0040
	Fe-Fe	6	2.85±0.01	0.0069		
Fe/SAs@Mo-based HNSs	Fe-O	5.9±0.3	1.98±0.01	0.0054	-6.3±1.6	0.0059

^aN: coordination numbers; ^bR: bond distance; $^c\sigma^2$: Debye-Waller factors; ^{d} ΔE_0: the inner potential correction. *R* factor: goodness of fit.

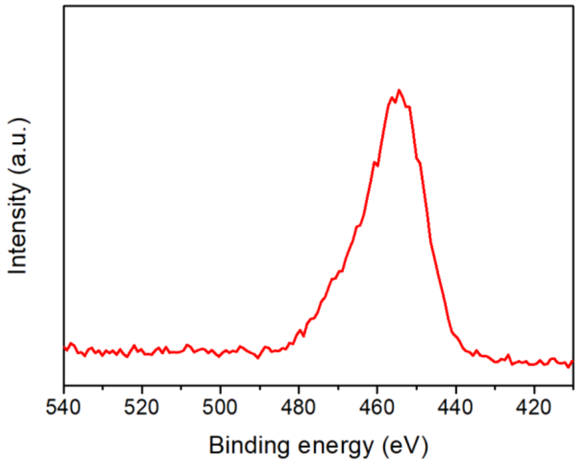

Supplementary Fig. 19 O 1*s* XPS spectra of Fe/SAs@Mo-based-HNSs.

Supplementary Table 5 Elemental compositions for FePMoG, FeMoP-450, Fe/SAs@Mo-based-HNSs, FeMoP-550 and tested FeMoP-500 determined by XPS.

Samples	Fe (at%)	Mo (at%)	P (at%)	O (at%)	$r_{Fe/Mo}$
FePMoG	7.8	16.9	0.4	74.9	0.461
FeMoP-450	4.5	18.8	8.1	68.6	0.239
Fe/SAs@Mo-based HNSs	4.6	20.7	10.4	64.3	0.222
FeMoP-550	1.0	17.3	19.8	61.9	0.058
Tested FeMoP-500	4.1	19.2	10.2	66.5	0.213

$r_{Fe/Mo}$ is the atomic ratio of Fe to Mo

Supplementary Table 6 Elemental compositions for FePMoG, FeMoP-450, Fe/SAs@Mo-based-HNSs and FeMoP-550 determined by TEM EDS.

Samples	Fe (at%)	Mo (at%)	P (at%)	O (at%)	$r_{Fe/Mo}$
FePMoG	8.28	15.56	0.51	75.65	0.532
FeMoP-450	5.3	19.7	8.9	66.1	0.269
Fe/SAs@Mo-based HNSs	5.1	21.4	11.3	62.2	0.238
FeMoP-550	1.4	18.2	21.9	58.5	0.077

$r_{Fe/Mo}$ is the atomic ratio of Fe to Mo.

Supplementary Table 7 Elemental compositions for FePMoG, FeMoP-450, Fe/SAs@Mo-based-HNSs and FeMoP-550 determined by ICP-OES.

Samples	Fe (mg/kg)	Mo (mg/kg)	P (mg/kg)	O (mg/kg)	$r_{Fe/Mo}$
FePMoG	146725.83	481927.19	/	/	0.523
FeMoP-450	70586.59	431511.55	/	/	0.281
Fe/SAs@Mo-based HNSs	62367.61	420141.41	/	/	0.255
FeMoP-550	22122.89	447094.81	/	/	0.085

$r_{Fe/Mo}$ is the atomic ratio of Fe to Mo.

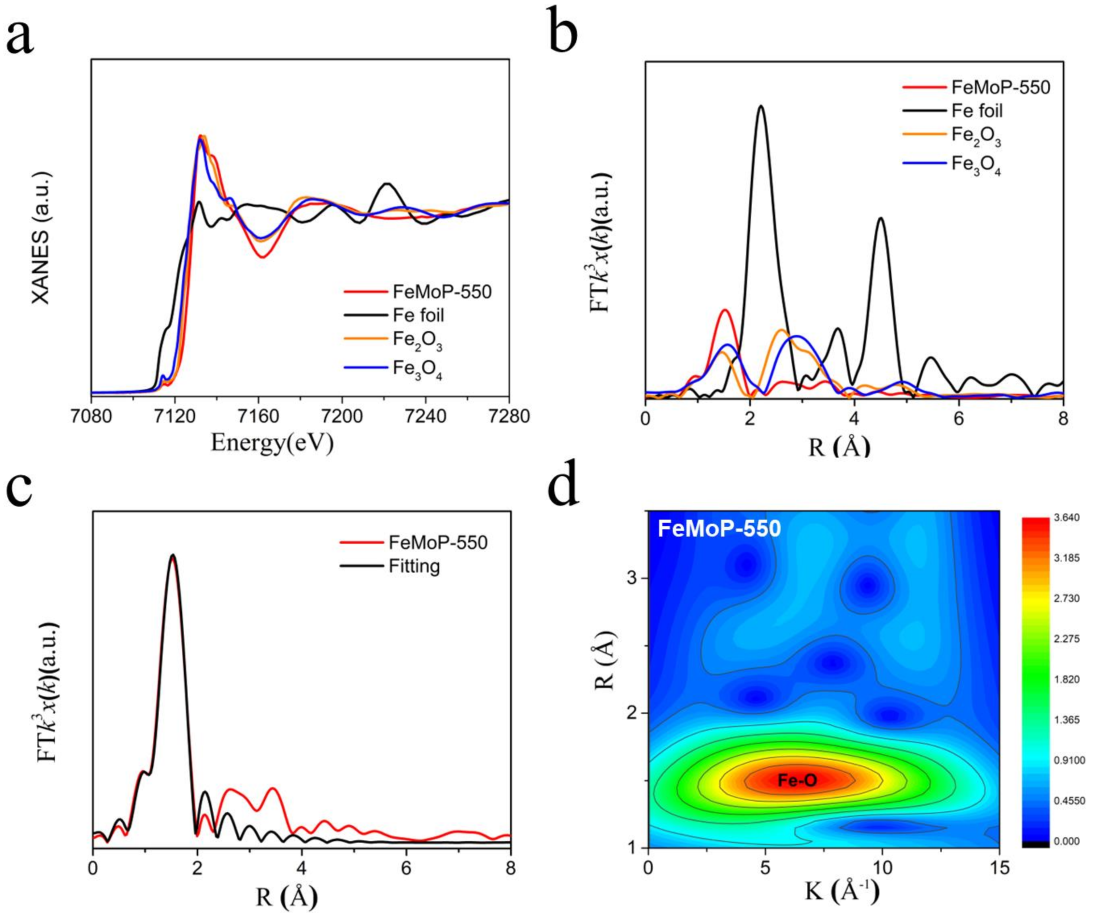

Supplementary Fig. 20 Spectroscopy of FeMoP-550 at Fe K-edge: (a) Fe K-edge XANES spectra; (b) corresponding k^3-weighted FT of EXAFS spectra; (c) the corresponding k^3-weighted FT-EXAFS spectra and fitting line in the R spacing; and (d) wavelet transforms for k^3-weighted EXAFS signals.

Supplementary Table 8 EXAFS fitting parameters at the Fe K-edge for various samples（S_0^2=0.74）

	shell	CN	R(Å)	σ^2	ΔE_0	R factor
Fe foil	Fe-Fe	8	2.47±0.01	0.0049	6.5±1.2	0.0066
	Fe-Fe	6	2.85±0.01	0.0060		
FeMoP-550	Fe-O	6.3±0.2	1.98±0.01	0.0045	-2.4±1.1	0.0039

[a]*N*: coordination numbers; [b]*R*: bond distance; [c]σ^2: Debye-Waller factors; [d] ΔE_0: the inner potential correction. *R* factor: goodness of fit.

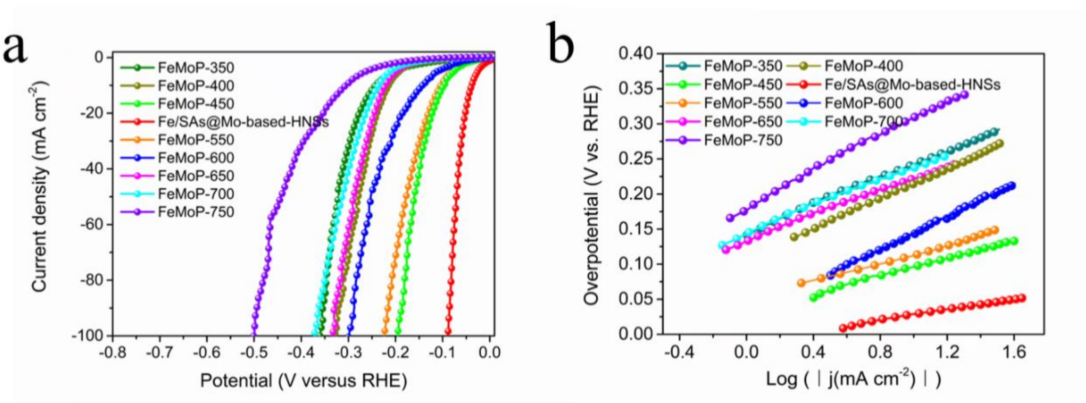

Supplementary Fig. 21 (a) Polarization curves of the FeMoP samples in 1 M KOH with iR correction. (b) Corresponding Tafel plots.

Supplementary Table 9 Comparison of HER performance in 1 M KOH for FeMoP-T catalysts.

Samples	η_{10} (mV)	Tafel slope (mV dec^{-1})
FeMoP-350	242.7	104.1
FeMoP-400	214.4	90.5
FeMoP-450	97.0	57.6
**Fe/SAs@Mo-based-HNSs (FeMoP-500)**	**38.5**	**35.6**
FeMoP-550	104.3	60.4
FeMoP-600	143.1	84.7
FeMoP-650	222.3	88.9
FeMoP-700	236.4	86.1
FeMoP-750	305.4	122.6
Bulk FeMoP-500	257.1	89.3

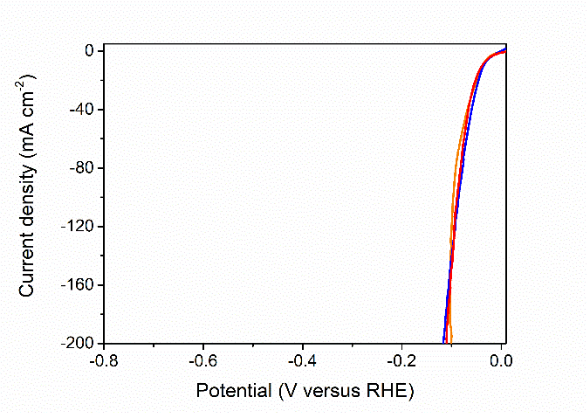

Supplementary Fig. 22 Polarization curves of three independent Fe/SAs@Mo-based-HNSs prepared from different batches in 1 M KOH with iR correction.

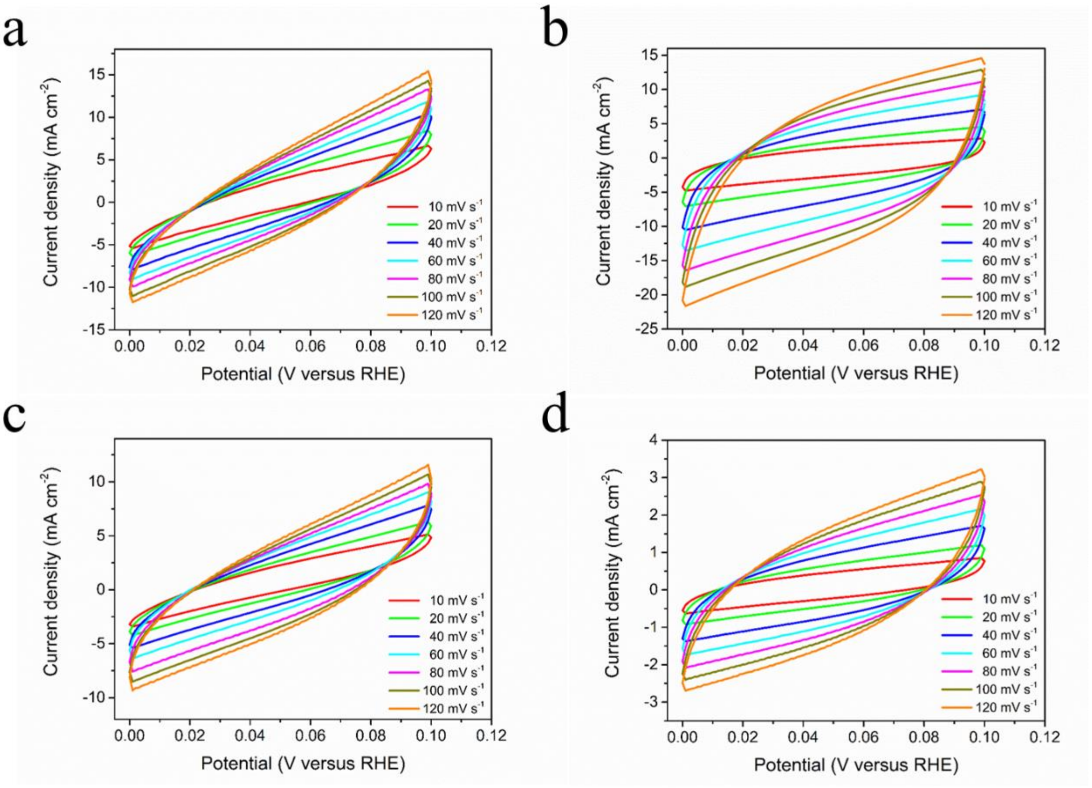

Supplementary Fig. 23 Cyclic voltammograms in the region of 0-0.1 V vs. RHE at various scan rates for (a) FeMoP-450, (b) Fe/SAs@Mo-based-HNSs, (c) FeMoP-550, and (d) Bulk FeMoP-500.

Supplementary Table 10 Amount of hydrogen production for samples at a constant current density of 10 mA/cm^2.*

Time (min)	Bulk FeMoP-500 (μmol)	20% Pt/C (μmol)	Fe/SAs@Mo-based-HNSs (μmol)
15	11.77±0. 60	20.40±1. 08	21.93±1. 06
30	23.20±0. 64	40.39±0. 89	44.89±0. 79
45	35.45±1. 05	60.59±0. 61	68.64±0. 81
60	47.08±0. 47	80.82±0. 54	88.80±0. 56
75	59.31±0. 46	101.16±0. 64	113.24±0. 78
90	71.99±0. 62	121.44±0. 88	134.22±0. 68
105	83.67±0. 31	140.92±0. 39	158.44±0. 78
120	94. 77±0. 52	162. 06±0. 47	179.46±0. 70

* This value is the average of three different electrode test results.

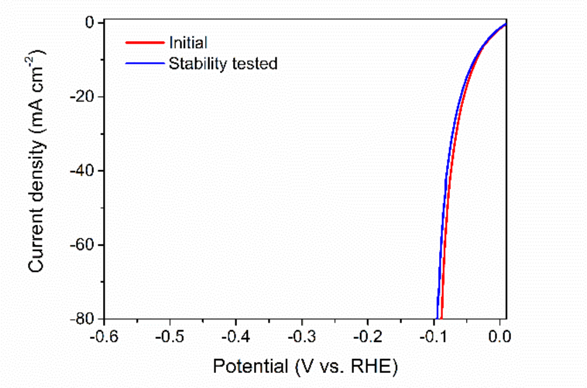

Supplementary Fig. 24 Polarisation curves of Fe/SAs@Mo-based-HNSs before and after 500 h test at a scan rate of 5 mV s^{-1} with potential error (iR) correction

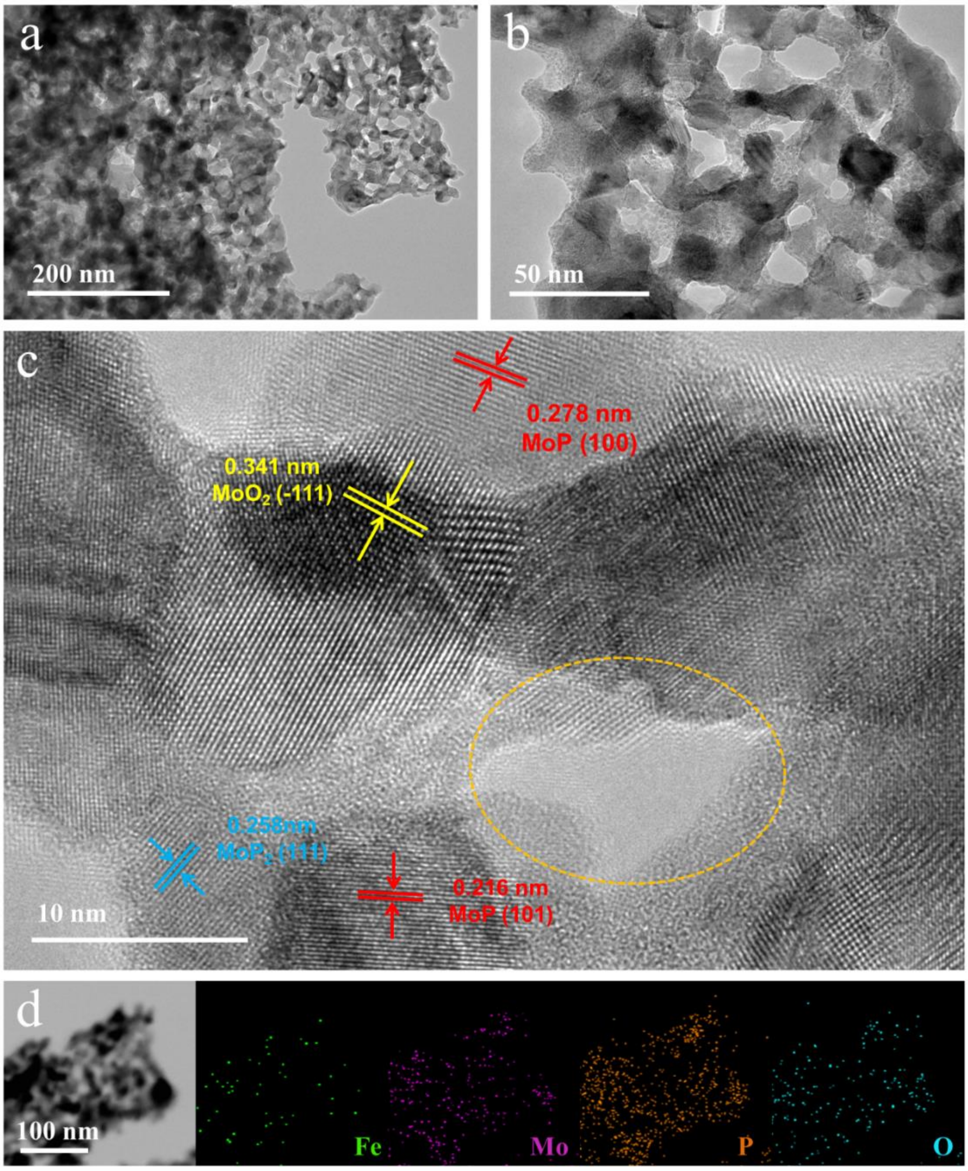

Supplementary Fig. 25 (a) Low magnification TEM image, (b) High magnification TEM image, (c) high-resolution TEM image, (d) STEM image and the corresponding EDS elemental mapping of Fe (green), Mo (violet), P (orange) and O (cyan) of the Fe/SAs@Mo-based-HNSs after 500 h test.

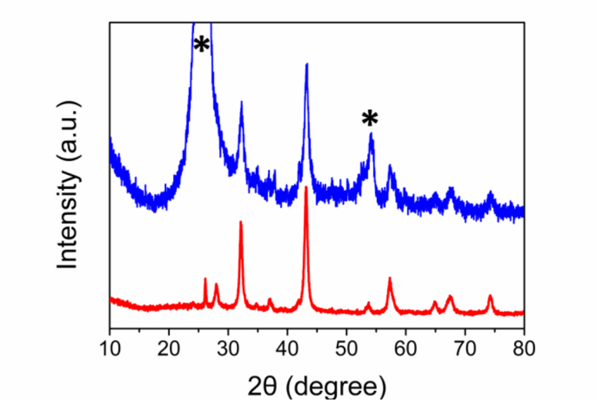

Supplementary Fig. 26 XRD pattern of the Fe/SAs@Mo-based-HNSs after 500 h test (blue curve). The peaks indexed with mark * derived from the carbon paper as the sample load on the carbon paper.

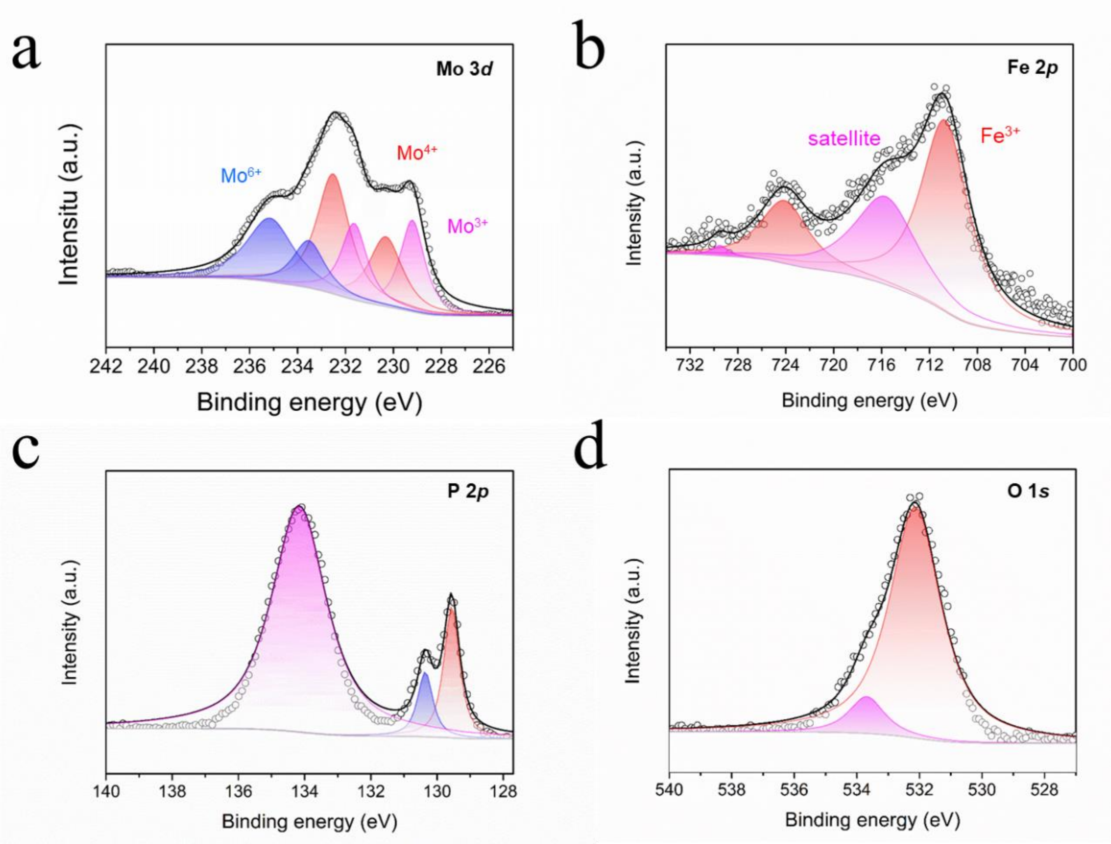

Supplementary Fig. 27 (a) Mo 3*d*, (b) Fe 2*p* spectrum, (c) P 2*p* spectrum, and (d) O 1*s* spectrum of the Fe/SAs@Mo-based-HNSs after 500 h test.

Supplementary Table 11 ICP-OES results of the Fe/SAs@Mo-based-HNSs after stability test for 500h at 20 mA cm^{-2} current density.

	Fe	Mo	P
Concentration (mmol/L)	0.014	0.009	0.012
Limit of reporting (mmol/L)	0.010	0.0058	0.030

**The lowest concentration of a substance that can be reliably reported by ICP-OES.*

Supplementary Table 12 Comparison of HER performance in 1 M KOH for Fe/SAs@Mo-based-HNSs and previous reported catalysts.

Electrocatalysts	Overpotential (at 10 mA cm^{-2})	Tafel slope (mV dec^{-1})	Loading (mg cm^{-2})	C_{dl} (mF cm^{-2})	Stability (h)	Ref.
**Mo-based**						
Fe/SAs@Mo-based-HNSs	39.5	47.8	0.67	40.3	600	This work
$Mo_2C/Mo_2N/C$-850	60	55	0.38	22.5	16.6	[25]
MoP@NCHSs-900	92	62	0.39	~130	12	[26]
FLNPC@MoP-NC	69	52	~5	55.3	50	[27]
MoP NA/CC	80	83	~2.5	4.02	46	[28]
MoP/SNG-20	49	31	0.4-0.5	20.5	20	[29]
**Fe-based**						
Ni-FeP/TiN/CC-1	75	73	N/A	33.7	10	[30]
NiFe LDH-NS@DG10	300	110	0.283	4.3	5.6	[31]
$FePSe_3$/NC	118.5	88	0.212	3.03	24	[32]
rGO-few layer $FePSe_3$	155	37	0.15	5.9	40	[33]
$FeNi_3N$/NG	~90	83.1	2	33.3	22	[34]
**Heterostructure**						
Mo_2N–Mo_2C/HGr	154	68	0.337	33	50	[35]
MoP/Ni_2P/NF	75	100.2	N/A	4.78*	24	[36]
1T-2H MoS_2	~290	65	0.28	2.41	28	[37]
MoS_2/Ni_3S_2	110	83.1	~7	15.6	10	[38]
$Ni(OH)_2/MoS_2$	80	60	4	735	17	[39]
$CoSe_2/MoSe_2$	218	76	0.204	4.3	N/A	[40]
HF-MoSP-900	119	85	0.07065	45.3	30	[41]
ES-WC/W_2C	75	59	0.075	97.8	20	[42]
W/W_2C@NPC-2	82	84.6	0.283	4.52	10	[43]
WC/W_2C@C NWs	56	52	0.34	61	45	[44]
CoP/NiCoP/NC	75	59	0.318	~19	85	[45]
CoP/NiCoP NTs	133	88	2	N/A	24	[46]
CoP-CoMo	198	95	1.12	N/A	10	[47]
$Co_2P/CoWO_4$	81	47	N/A	21.72	16	[48]
$Ni(OH)_2$-Ni_3N/TM	~62	86	3.2	7.25	25	[49]
$NiCo_2S_4/Ni_3S_2$/Ni	119	105.2	N/A	113.5	24	[50]
Ni/Ni_8P_3	130	58.5	N/A	23.1	24	[51]
Ni_2P-$NiSe_2$/CC	66	72.6	9.2	31	12	[52]
**Single atoms**						
Pt_1/N-C	46	36.8	0.25	N/A	20	[53]
Fe_1/NC	111.1	86.1	~0.3	N/A	20	[54]
Pt_1@Fe-N-C	108	~50	0.4	N/A	20	[55]
Co_1N_x/C	247	75	2	N/A	N/A	[56]
Ru-MoS_2/CC	41	114	12.446	N/A	20	[57]
Pt_1/PCM	139	73.6	21.23	51.09	5	[58]
Co1/PCN	89	59	0.5	N/A	24	[59]
Mo_1/N_1C_2	132	90	0.408	N/A	N/A	[60]
E-Co SAs	59	105	0.4	N/A	500	[61]
**2D nanosheets**						
WS_2/rGO	~250	58	0.4	N/A	N/A	[62]

$Co_2B/CoSe_2$	320	76	0.4	36.2	30	[63]
$MoP@Ni_3P/NF$	45	56	N/A	7.7	35	[64]
CoP NS/CNTs	68	57	0.272	3.2	24	[65]
Ni-BDT-A	80	70	N/A	59.2	N/A	[66]
**Noble-metal-based**						
4H/fcc Au-Ru-2 NWs	50	30.8	0.08	N/A	N/A	[67]
20% $Pt/Ni(HCO_3)_2$	27	45	0.204	2.58	30.5	[68]
$Pt–Co(OH)_2/CC$	32	70	6.9	68	20	[69]
w-Au@MoS2	120	52.9	N/A	N/A	10	[70]
$Ni@Ni_2P–Ru$	31	41	0.283	N/A	11	[71]
Pd-Pt-S	71	31	$\sim 0.02_{Pd+Pt}$	N/A	2	[72]
**Multiple metal-based**						
$VN@Ni_3N–Ni-6/CC$	57	40	N/A	31.86	40	[73]
Co_4Ni_1P NTs	129	~460	0.19	52	20	[74]
$P-Co_3Mo_3C/Co/CNF$	81	64	0.43	35	40	[75]
Ni–P/MoSx	140	64	N/A	3.5	10	[76]
TiO_2 NDs/Co NSNTs-CFs	~90	62	N/A	71.1	100	[77]
$MoS_2\|NiS\|MoO_3$	91	54.5	N/A	45.12	20	[78]
$Co_3S_4@MoS_2$	136	74	0.283	62	10	[79]
Cu NDs/Ni_3S_2 NTs-CFs	128	76.2	N/A	62.37	32	[80]
$Cu@CoS_x/CF$	270	61	N/A	N/A	200	[81]
np-$(Co_{0.52}Fe_{0.48})_2P$	~70	40	1	N/A	50	[82]
2D-$MoS_2/Co(OH)_2$	128	76	0.285	0.91	20	[83]
NiS_2/MoS_2 HNW	204	65	0.2	5.6	6	[84]
H-$CeO_{2-x}/Ni_2P@NC$	123	60	0.35	21.76	27	[85]
carbon-free						
MoP/NF	114	54.6	N/A	52.1	20	[86]
MoP NSAs/Mo	106	55	~1	73	36	[87]
Ni_2P NPs	140	~30	1	N/A	200	[88]
FeP NAs/CC	58	146	1.5	60	20	[89]
Co/Co_3O_4 NS	~55	44	0.85	N/A	2	[90]
CoP/CC	67	51	0.92	N/A	22.2	[91]

**Obtained from the figures in the reference.*

Supplementary Table 13 The H_2O adsorption energy, adsorption site and the bonding distance between active site and O atom in H_2O.

Catalyst	Adsorption energy (eV)	Adsorption site	Mo/Fe-O bonding distance
MoP	-0.64	Mo top	2.334 Å
MoP_2	-0.62	Mo top	2.410 Å
MoO_2	-0.79	Mo top	2.284 Å
MoP/MoP_2	-0.99	Mo top	2.187 Å
MoP/MoO_2	-0.88	Mo top	2.439 Å
$MoP2/MoO_2$	-0.94	Mo top	2.410 Å
Fe@MoO_2-1	-0.92	Fe top	2.027 Å
Fe@MoO_2-2	-0.36	Fe top	2.271 Å

Supplementary Table S13 lists the H_2O adsorption energies, active adsorption sites, and the bonding distance between the O atoms of H_2O and atoms in electrocatalytic active sites for all of the models. It can be seen that the H_2O adsorption ability of single-phase models is inversely proportional to the Mo–O bonding distance, which is consistent with H_2O adsorption abilities varying with Mo–O and Fe–O bonding distances in heterostructured interface models and monoatomic dispersed Fe models. Intriguingly, even though the Mo–O bonding distances for MoP/MoO_2 and MoP_2/MoO_2 (2.439 Å and 2.410 Å, respectively) are larger than those for MoP, MoP_2, and MoO_2 (2.334 Å, 2.410 Å, and 2.284 Å, respectively), the H_2O adsorption ability of heterostructured interfaces is higher than that of the single phase. This demonstrates that heterostructured interfaces in Fe SAs@Mo-based HNSs effectively promote the transfer of electrons from active sites to H_2O molecules, and thus improve these compounds' H_2O adsorption ability.

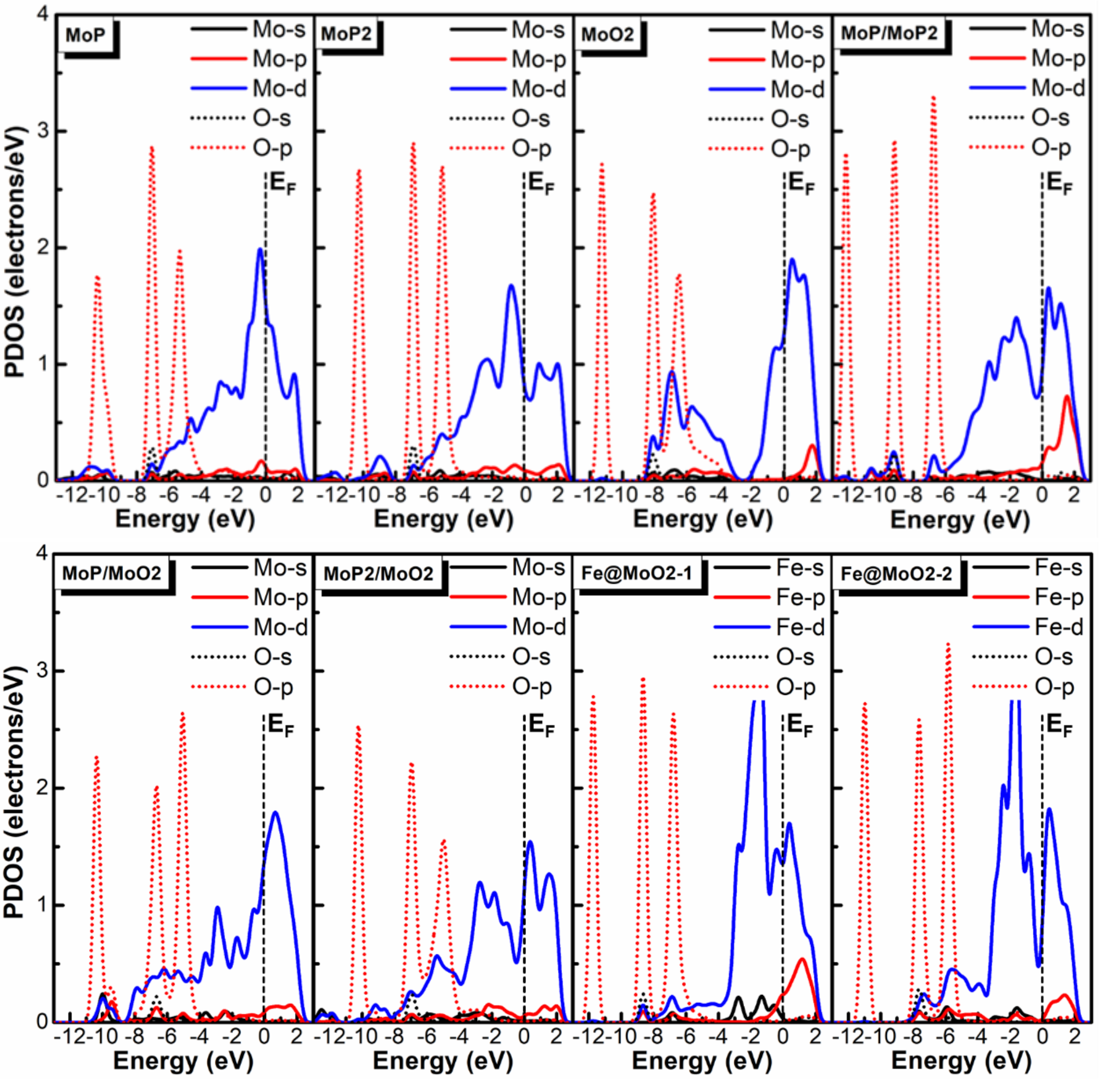

Supplementary Fig. 28 Partial density of states (PDOS) of MoP, MoP_2, MoO_2, MoP/MoP_2, MoP/MoO_2, MoP_2/MoO_2, Fe@MoO_2-1 and Fe@MoO_2-2 after H_2O adsorption at surface sites. The black dashed lines at the energy of zero indicate the Fermi level (E_F).

To further explore the strength of bonding between H_2O molecules and active sites, we determined the partial density of states (PDOS) curves of Mo/Fe active sites and O atoms in H_2O molecules (Supplementary Fig. 28). As can be seen, the intensities of interaction between the electrons in the s-, p-, d-orbitals of Mo/Fe and the s-, p-orbitals of O are rather different, which accounts for the variation in the H_2O adsorption ability of different models. The PDOS peak overlap of Mo and O orbitals is most extensive in MoO_2 in the single-phase models, whereas it is most extensive in MoP/MoP_2 in the heterostructured interface models. In addition, the PDOS peaks of O perfectly overlap with that of Fe in Fe@MoO_2-1 but not in Fe@MoO_2-2, which accounts for the significant difference in these species' H_2O adsorption ability. These physical insights explain the variation in the electron-transfer ability of different structures, and thus highlight the microstructures that are critical for efficient H_2O adsorption.

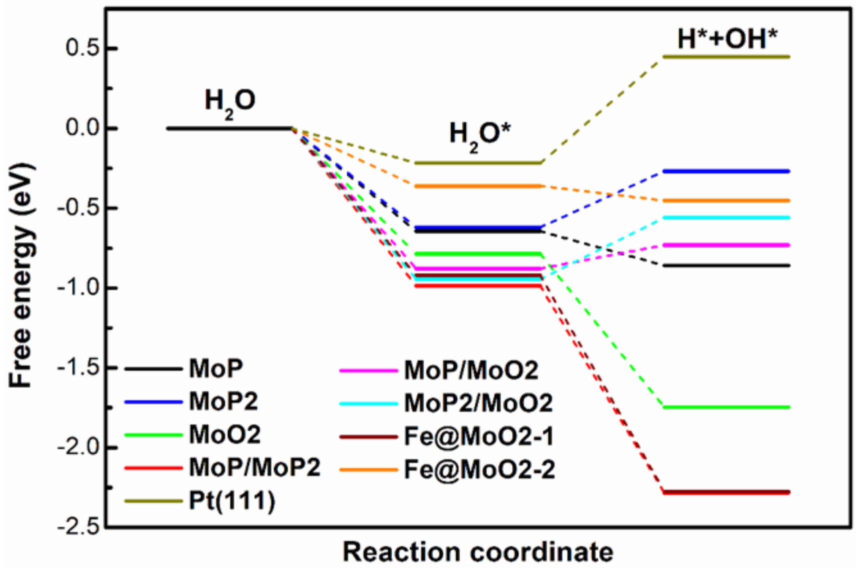

Supplementary Fig. 29 Free energy diagrams of reaction coordinate for water dissociation on the surfaces of single-phase models (MoP, MoP_2 and MoO_2), heterostructured interface models (MoP/MoP_2, MoP/MoO_2 and MoP_2/MoO_2), monoatomic dispersed Fe onto MoO_2 surface models (Fe@MoO_2-1 and Fe@MoO_2-2) and Pt(111).

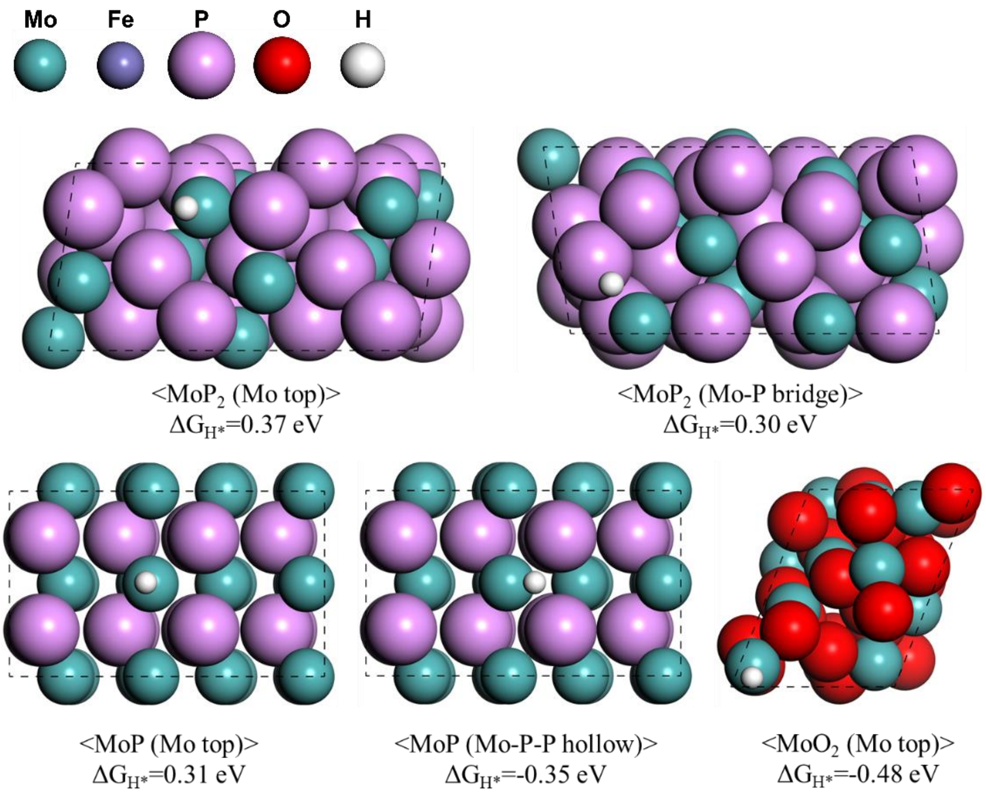

Supplementary Fig. 30 The representative atomic configurations after H adsorption at the surface sites of MoP, MoP_2 and MoO_2 with corresponding ΔG_{H*}.

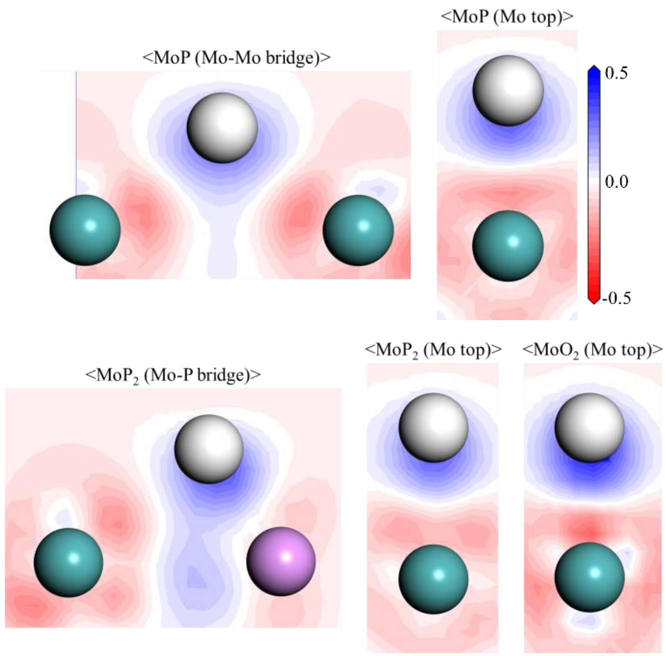

Supplementary Fig. 31 DFT results of 2D electron density differences after adsorption of H* onto the active sites in the single-phase models. Red and blue represent the depletion and accumulation of electrons with the unit of e/Å^3, respectively.

In the single-phase models (Supplementary Fig. 30), it can be seen that electron transfer between H* and Mo is too weak at the Mo top of MoP, given its ΔG_{H^*} value of 0.31 eV. A stronger depletion of electrons in Mo occurs at the Mo–Mo bridge than that at the Mo top, which improves H* adsorption at the surface of MoP; however, this interaction is too strong, resulting in an unsuitable ΔG_{H^*} value (-0.35 eV). The electron-transfer ability of the Mo top of MoP_2 is similar to that of MoP, which results in a similarly unsuitable ΔG_{H^*} value (0.37 eV). The Mo–P bridge in MoP_2 shows a slightly stronger interaction with H*, owing to additional electron transfer from P to H*, and thus its ΔG_{H^*} value is slightly better (0.30 eV) than those of the Mo–Mo bridge and the Mo top of MoP. Compared with <MoP (Mo top)> and <MoO_2 (Mo top)>, the ability of Mo to transfer electrons to H* is much stronger in MoO_2; however, it is too strong, as it results in a ΔG_{H^*} value of -0.48 eV. While in the heterostructured interface models (Figure 5e), the electron transfer from the MoP/MoP_2 interface to H* is too high, leading to a ΔG_{H^*} value of -0.37 eV; in contrast, the electron transfer from the MoP/MoO_2 interface to H* is much lower, leading to a better (i.e., closer to zero) ΔG_{H^*} value (0.22 eV).

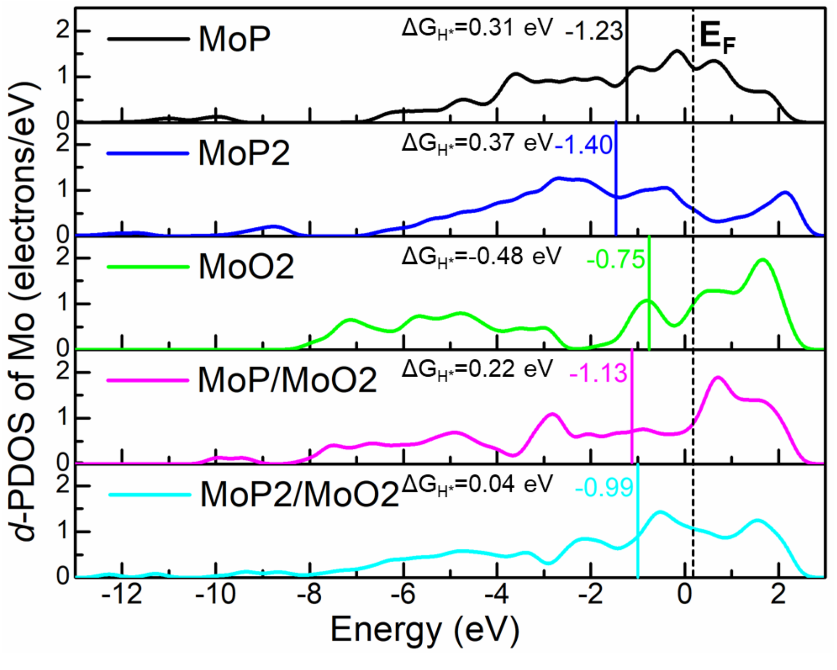

Supplementary Fig. 32 The *d*-orbital partial density of states (*d*-PDOS) of three single-phase and two heterostructured interface models with H adsorption onto Mo top. The solid lines marked with digitals show the position of d-band centers and the dashed line indicate Fermi level (E_F). The corresponding ΔG_{H^*} values for the models are also indicated.

To ensure the comparability of d-band centres in active sites, we examined only the same Mo

top sites in single-phase and heterostructured interface models: thus, MoP/MoO_2 and MoP_2/MoO_2 interface models with optimal ΔG_{H*} values were compared with the single-phase models

Supplementary Table 14 The ΔG_{H*} and *d*-band centers of three single-phase and two heterostructured interface models with H adsorption onto Mo top.

	MoP_2	MoP	MoP/MoO_2	MoP_2/MoO_2	MoO_2
ΔG_{H*} (eV)	0.37	0.31	0.22	0.04	-0.48
d-band center	-1.40	-1.23	-1.13	-0.99	-0.75

References

1 Lin, C. *et al.* 2D-organic framework confined metal single atoms with the loading reaching the theoretical limit. *Mater. Horiz.* **7**, 2726-2733 (2020).

2 Li, P. *et al.* Nickel single atom-decorated carbon nanosheets as multifunctional electrocatalyst supports toward efficient alkaline hydrogen evolution. *Nano Energy* **83**, 105850 (2021).

3 Wu, S. *et al.* Highly durable organic electrode for sodium-ion batteries via a stabilized α-C radical intermediate. *Nat. Commun.* **7**, 1-11 (2016).

4 Bulushev, D. A. *et al.* Ni-N4 sites in a single-atom Ni catalyst on N-doped carbon for hydrogen production from formic acid. *J. Catal.* **402**, 264-274 (2021).

5 Peng, R.-L. *et al.* Single-atom implanted two-dimensional MOFs as efficient electrocatalysts for the oxygen evolution reaction. *Inorg. Chem. Front.* **7**, 4661-4668 (2020).

6 Li, B. *et al.* Mineral Hydrogel from Inorganic Salts: Biocompatible Synthesis, All-in-One Charge Storage, and Possible Implications in the Origin of Life. *Adv. Funct. Mater.* **32**, 2109302 (2022).

7 Dong, J., Lv, C., Humphrey, M. G., Zhang, C. & Huang, Z. One-dimensional amorphous cobalt (II) metal–organic framework nanowire for efficient hydrogen evolution reaction. *Inorg. Chem. Front.* (2022).

8 Liu, B. *et al.* Hybrid heterojunction of molybdenum disulfide/single cobalt atoms anchored nitrogen, sulfur-doped carbon nanotube/cobalt disulfide with multiple active sites for highly efficient hydrogen evolution. *Appl. Catal., B* **298**, 120630 (2021).

9 Tavakkoli, M. *et al.* Electrochemical activation of single-walled carbon nanotubes with pseudo-atomic-scale platinum for the hydrogen evolution reaction. *ACS Catal.* **7**, 3121-3130 (2017).

10 Sun, Y. *et al.* Modulating electronic structure of metal-organic frameworks by introducing atomically dispersed Ru for efficient hydrogen evolution. *Nat. Commun.* **12**, 1-8 (2021).

11 Yin, X. P. *et al.* Engineering the coordination environment of single-atom platinum anchored on graphdiyne for optimizing electrocatalytic hydrogen evolution. *Angew. Chem. Int. Ed.* **57**, 9382-9386 (2018).

12 Subrahmanyam, K. S. *et al.* High-surface-area antimony sulfide chalcogels. *Chem. Mater.* **28**, 7744-7749 (2016).

13 Bag, S., Gaudette, A. F., Bussell, M. E. & Kanatzidis, M. G. Spongy chalcogels of non-platinum metals act as effective hydrodesulfurization catalysts. *Nat. Chem.* **1**, 217-224 (2009).

14 Mondal, C. *et al.* Pure inorganic gel: a new host with tremendous sorption capability. *Chem. Commun.* **49**, 9428-9430 (2013).

15 Feng, H. *et al.* Porphyrin-based Ti-MOFs conferred with single-atom Pt for enhanced photocatalytic hydrogen evolution and NO removal. *Chem. Eng. J.* **428**, 132045 (2022).

16 Cao, B. *et al.* Tailoring the d-band center of N-doped carbon nanotube arrays with Co4N nanoparticles and single-atom Co for a superior hydrogen evolution reaction. *NPG Asia Mater.* **13**, 1-14 (2021).

17 Jiao, L. & Jiang, H.-L. Metal-organic-framework-based single-atom catalysts for energy applications. *Chem* **5**, 786-804 (2019).

18 Dong, P. *et al.* Platinum single atoms anchored on a covalent organic framework: boosting active sites for photocatalytic hydrogen evolution. *ACS Catal.* **11**, 13266-13279 (2021).

19 Wang, J., Wang, J., Qi, S. & Zhao, M. Stable multifunctional single-atom catalysts resulting from the synergistic effect of anchored transition-metal atoms and host covalent–organic frameworks. *J. Phys. Chem. C* **124**, 17675-17683 (2020).

20 Ji, Y., Dong, H., Liu, C. & Li, Y. Two-dimensional π-conjugated metal–organic nanosheets as single-atom catalysts for the hydrogen evolution reaction. *Nanoscale* **11**, 454-458 (2019).

21 Zhu, Z. *et al.* Coexisting single-atomic Fe and Ni sites on hierarchically ordered porous carbon as a highly efficient ORR electrocatalyst. *Adv. Mater.* **32**, 2004670 (2020).

22 Liu, R. *et al.* Enhanced proton and electron reservoir abilities of polyoxometalate grafted on graphene for high-performance hydrogen evolution. *Energy Environ. Sci.* **9**, 1012-1023 (2016).

23 Tan, C. Self-assembly, aggregates morphology and ionic liquid crystal of polyoxometalate-based hybrid molecule: From vesicles to layered structure. *J. Mol. Struct.***1148**, 34-39 (2017).

24 Zhai, Q. *et al.* A novel iron-containing polyoxometalate heterogeneous photocatalyst for efficient 4-chlorophennol degradation by H2O2 at neutral pH. *Appl. Surf. Sci.* **377**, 17-22 (2016).

25 Li, S. *et al.* Metal-organic precursor–derived mesoporous carbon spheres with homogeneously distributed molybdenum carbide/nitride nanoparticles for efficient hydrogen evolution in alkaline media. *Adv. Funct. Mater.* **29**, 1807419 (2019).

26 Zhao, D. *et al.* Synergistically Interactive Pyridinic-N–MoP Sites: Identified Active Centers for Enhanced Hydrogen Evolution in Alkaline Solution. *Angew. Chem. Int. Ed.* **132**, 9067-9075 (2020).

27 Liu, B. *et al.* Few Layered N, P Dual-Doped Carbon-Encapsulated Ultrafine MoP Nanocrystal/MoP Cluster Hybrids on Carbon Cloth: An Ultrahigh Active and Durable 3D Self-Supported Integrated Electrode for Hydrogen Evolution Reaction in a Wide pH Range. *Adv. Funct. Mater.* **28**, 1801527 (2018).

28 Pu, Z., Wei, S., Chen, Z. & Mu, S. Flexible molybdenum phosphide nanosheet array electrodes for hydrogen evolution reaction in a wide pH range. *Appl. Catal., B* **196**, 193-198 (2016).

29 Anjum, M. A. R. & Lee, J. S. Sulfur and nitrogen dual-doped molybdenum phosphide

nanocrystallites as an active and stable hydrogen evolution reaction electrocatalyst in acidic and alkaline media. *ACS Catal.* **7**, 3030-3038 (2017).

30 Peng, X. *et al.* Ni-doped amorphous iron phosphide nanoparticles on TiN nanowire arrays: An advanced alkaline hydrogen evolution electrocatalyst. *Nano Energy* **53**, 66-73 (2018).

31 Jia, Y. *et al.* A heterostructure coupling of exfoliated Ni–Fe hydroxide nanosheet and defective graphene as a bifunctional electrocatalyst for overall water splitting. *Adv. Mater.* **29**, 1700017 (2017).

32 Yu, J. *et al.* Metallic FePSe3 nanoparticles anchored on N-doped carbon framework for All-pH hydrogen evolution reaction. *Nano Energy* **57**, 222-229 (2019).

33 Mukherjee, D. & Sampath, S. Few-layer iron selenophosphate, FePSe3: efficient electrocatalyst toward water splitting and oxygen reduction reactions. *ACS Appl. Energy Mater.* **1**, 220-231 (2017).

34 Liu, L. *et al.* Ultrasmall FeNi 3 N particles with an exposed active (110) surface anchored on nitrogen-doped graphene for multifunctional electrocatalysts. *J. Mater. Chem. A* **7**, 1083-1091 (2019).

35 Yan, H. *et al.* Holey reduced graphene oxide coupled with an Mo2N–Mo2C heterojunction for efficient hydrogen evolution. *Adv. Mater.* **30**, 1704156 (2018).

36 Du, C., Shang, M., Mao, J. & Song, W. Hierarchical MoP/Ni 2 P heterostructures on nickel foam for efficient water splitting. *J. Mater. Chem. A* **5**, 15940-15949 (2017).

37 Wang, S. *et al.* Ultrastable In-Plane 1T–2H MoS2 Heterostructures for Enhanced Hydrogen Evolution Reaction. *Adv. Energy. Mater.* **8**, 1801345 (2018).

38 Zhang, J. *et al.* Interface engineering of MoS2/Ni3S2 heterostructures for highly enhanced electrochemical overall-water-splitting activity. *Angew. Chem. Int. Ed.* **55**, 6702-6707 (2016).

39 Zhang, B. *et al.* Interface engineering: the Ni (OH) 2/MoS2 heterostructure for highly efficient alkaline hydrogen evolution. *Nano Energy* **37**, 74-80 (2017).

40 Zhao, G. *et al.* CoSe2/MoSe2 Heterostructures with enriched water adsorption/dissociation sites towards enhanced alkaline hydrogen evolution reaction. *Chem. - Eur. J.* **24**, 11158-11165 (2018).

41 Wu, A. *et al.* Hierarchical MoS 2@ MoP core–shell heterojunction electrocatalysts for efficient hydrogen evolution reaction over a broad pH range. *Nanoscale* **8**, 11052-11059 (2016).

42 Chen, Z. *et al.* Eutectoid-structured WC/W2C heterostructures: A new platform for long-term alkaline hydrogen evolution reaction at low overpotentials. *Nano Energy* **68**, 104335 (2020).

43 Zhang, Q. *et al.* A robust electrocatalytic activity toward the hydrogen evolution reaction from W/W 2 C heterostructured nanoparticles coated with a N, P dual-doped carbon layer. *Chem. Commun.* **55**, 9665-9668 (2019).

44 Zhang, L.-N. *et al.* Ultrafine cable-like WC/W 2 C heterojunction nanowires covered by graphitic carbon towards highly efficient electrocatalytic hydrogen evolution. *J. Mater. Chem. A* **6**, 15395-15403 (2018).

45 Boppella, R., Tan, J., Yang, W. & Moon, J. Homologous CoP/NiCoP Heterostructure on N-Doped Carbon for Highly Efficient and pH-Universal Hydrogen Evolution Electrocatalysis. *Adv. Funct. Mater.* **29**, 1807976 (2019).

46 Lin, Y. *et al.* Construction of CoP/NiCoP Nanotadpoles Heterojunction Interface for Wide pH Hydrogen Evolution Electrocatalysis and Supercapacitor. *Adv. Energy Mater.* **9**, 1901213 (2019).

47 Yu, L. *et al.* Cobalt/Molybdenum Phosphide and Oxide Heterostructures Encapsulated in N-Doped Carbon Nanocomposite for Overall Water Splitting in Alkaline Media. *ACS Appl. Mater. Interfaces* **11**, 6890-6899 (2019).

48 Tao, K., Dan, H., Hai, Y., Liu, L. & Gong, Y. Ultrafine Co2P anchored on porous CoWO4 nanofiber matrix for hydrogen evolution: Anion-induced compositional/morphological transformation and interfacial electron transfer. *Electrochim. Acta* **328**, 135123 (2019).

49 Gao, M., Chen, L., Zhang, Z., Sun, X. & Zhang, S. Interface engineering of the Ni (OH) 2–Ni 3 N nanoarray heterostructure for the alkaline hydrogen evolution reaction. *J. Mater. Chem. A* **6**, 833-836 (2018).

50 Liu, H. *et al.* Heteromorphic NiCo2S4/Ni3S2/Ni foam as a self-standing electrode for hydrogen evolution reaction in alkaline solution. *ACS Appl. Mater. Interfaces* **10**, 10890-10897 (2018).

51 Chen, G. F. *et al.* Efficient and stable bifunctional electrocatalysts Ni/NixMy (M= P, S) for overall water splitting. *Adv. Funct. Mater.* **26**, 3314-3323 (2016).

52 Liu, C. *et al.* Engineering Ni2P-NiSe2 heterostructure interface for highly efficient alkaline hydrogen evolution. *Appl. Catal., B* **262**, 118245 (2020).

53 Fang, S. *et al.* Uncovering near-free platinum single-atom dynamics during electrochemical hydrogen evolution reaction. *Nat. Commun.* **11**, 1-8 (2020).

54 Wang, L. *et al.* Active Sites of Single-Atom Iron Catalyst for Electrochemical Hydrogen Evolution. *J. Phys. Chem. Lett.* **11**, 6691-6696 (2020).

55 Zeng, X. *et al.* Single-Atom to Single-Atom Grafting of Pt1 onto Fe N4 Center: Pt1@ Fe N C Multifunctional Electrocatalyst with Significantly Enhanced Properties. *Adv. Energy Mater.* **8**, 1701345 (2018).

56 Liang, H.-W. *et al.* Molecular metal–N x centres in porous carbon for electrocatalytic hydrogen evolution. *Nat. Commun.* **6**, 1-8 (2015).

57 Wang, D., Li, Q., Han, C., Xing, Z. & Yang, X. Single-atom ruthenium based catalyst for enhanced hydrogen evolution. *Appl. Catal., B* **249**, 91-97 (2019).

58 Zhang, H. *et al.* Dynamic traction of lattice-confined platinum atoms into mesoporous carbon matrix for hydrogen evolution reaction. *Sci. Adv.* **4**, eaao6657 (2018).

59 Cao, L. *et al.* Identification of single-atom active sites in carbon-based cobalt catalysts during electrocatalytic hydrogen evolution. *Nat. Catal.* **2**, 134-141 (2019).

60 Chen, W. *et al.* Rational design of single molybdenum atoms anchored on N-doped carbon for effective hydrogen evolution reaction. *Angew. Chem. Int. Ed.* **56**, 16086-16090 (2017).

61 Liu, X. *et al.* Identifying the Activity Origin of a Cobalt Single-Atom Catalyst for Hydrogen Evolution Using Supervised Learning. *Adv. Funct. Mater.* **31**, 2100547 (2021).

62 Yang, J. *et al.* Two-dimensional hybrid nanosheets of tungsten disulfide and reduced graphene oxide as catalysts for enhanced hydrogen evolution. *Angew. Chem. Int. Ed.* **52**, 13751-13754 (2013).

63 Guo, Y., Yao, Z., Shang, C. & Wang, E. Amorphous Co2B grown on CoSe2 nanosheets as a hybrid catalyst for efficient overall water splitting in alkaline medium. *ACS Appl. Mater. Interfaces* **9**, 39312-39317 (2017).

64 Wang, F. *et al.* Increased nucleation sites in nickel foam for the synthesis of MoP@ Ni3P/NF nanosheets for bifunctional water splitting. *Appl. Surf. Sci.* **481**, 1403-1411 (2019).
65 Zhang, Y. *et al.* Heterostructure of 2D CoP nanosheets/1D carbon nanotubes to significantly boost the alkaline hydrogen evolution. *Adv. Mater. Interfaces* **7**, 1901302 (2020).
66 Hu, C. *et al.* In situ electrochemical production of ultrathin nickel nanosheets for hydrogen evolution electrocatalysis. *Chem* **3**, 122-133 (2017).
67 Lu, Q. *et al.* Crystal phase-based epitaxial growth of hybrid noble metal nanostructures on 4H/fcc Au nanowires. *Nat. Chem.* **10**, 456-461 (2018).
68 Lao, M. *et al.* Platinum/nickel bicarbonate heterostructures towards accelerated hydrogen evolution under alkaline conditions. *Angew. Chem. Int. Ed.* **58**, 5432-5437 (2019).
69 Xing, Z., Han, C., Wang, D., Li, Q. & Yang, X. Ultrafine Pt nanoparticle-decorated Co (OH) 2 nanosheet arrays with enhanced catalytic activity toward hydrogen evolution. *ACS Catal.* **7**, 7131-7135 (2017).
70 Li, Y. *et al.* Morphological engineering of winged Au@ MoS2 heterostructures for electrocatalytic hydrogen evolution. *Nano lett.* **18**, 7104-7110 (2018).
71 Liu, Y. *et al.* Ru modulation effects in the synthesis of unique rod-like Ni@ Ni2P–Ru heterostructures and their remarkable electrocatalytic hydrogen evolution performance. *J. Am. Chem. Soc.* **140**, 2731-2734 (2018).
72 Fan, J. *et al.* Engineering Pt/Pd interfacial electronic structures for highly efficient hydrogen evolution and alcohol oxidation. *ACS Appl. Mater. Interfaces* **9**, 18008-18014 (2017).
73 Dong, X. *et al.* 3D hierarchical V–Ni-based nitride heterostructure as a highly efficient pH-universal electrocatalyst for the hydrogen evolution reaction. *J. Mater. Chem. A* **7**, 15823-15830 (2019).
74 Yan, L. *et al.* Metal-Organic Frameworks Derived Nanotube of Nickel–Cobalt Bimetal Phosphides as Highly Efficient Electrocatalysts for Overall Water Splitting. *Adv. Funct. Mater.* **27**, 1703455 (2017).
75 Qiu, L. *et al.* Phosphorus-doped Co3Mo3C/Co/CNFs hybrid: A remarkable electrocatalyst for hydrogen evolution reaction. *Electrochim. Acta* **325**, 134962 (2019).
76 Han, G.-Q. *et al.* Electrodeposited hybrid Ni–P/MoSx film as efficient electrocatalyst for hydrogen evolution in alkaline media. *Int. J. Hydrogen Energy* **42**, 2952-2960 (2017).
77 Feng, J. X. *et al.* Efficient hydrogen evolution electrocatalysis using cobalt nanotubes decorated with titanium dioxide nanodots. *Angew. Chem. Int. Ed.* **56**, 2960-2964 (2017).
78 Wang, C., Tian, B., Wu, M. & Wang, J. Revelation of the excellent intrinsic activity of MoS2| NiS| MoO3 nanowires for hydrogen evolution reaction in alkaline medium. *ACS Appl. Mater. Interfaces* **9**, 7084-7090 (2017).
79 Guo, Y. *et al.* Elaborately assembled core-shell structured metal sulfides as a bifunctional catalyst for highly efficient electrochemical overall water splitting. *Nano Energy* **47**, 494-502 (2018).
80 Feng, J.-X., Wu, J.-Q., Tong, Y.-X. & Li, G.-R. Efficient hydrogen evolution on Cu nanodots-decorated Ni3S2 nanotubes by optimizing atomic hydrogen adsorption and desorption. *J. Am. Chem. Soc.* **140**, 610-617 (2018).
81 Liu, Y. *et al.* Coupling Sub-Nanometric Copper Clusters with Quasi-Amorphous Cobalt Sulfide Yields Efficient and Robust Electrocatalysts for Water Splitting Reaction. *Adv.*

Mater. **29**, 1606200 (2017).

82 Tan, Y. *et al.* Versatile nanoporous bimetallic phosphides towards electrochemical water splitting. *Energy Environ. Sci.* **9**, 2257-2261 (2016).

83 Zhu, Z. *et al.* Ultrathin transition metal dichalcogenide/3d metal hydroxide hybridized nanosheets to enhance hydrogen evolution activity. *Adv. Mater.* **30**, 1801171 (2018).

84 Kuang, P., Tong, T., Fan, K. & Yu, J. In situ fabrication of Ni–Mo bimetal sulfide hybrid as an efficient electrocatalyst for hydrogen evolution over a wide pH range. *ACS Catal.* **7**, 6179-6187 (2017).

85 Zhang, B. *et al.* Strongly coupled hollow-oxide/phosphide hybrid coated with nitrogen-doped carbon as highly efficient electrocatalysts in alkaline for hydrogen evolution reaction. *J. Catal.* **377**, 582-588 (2019).

86 Jiang, Y., Lu, Y., Lin, J., Wang, X. & Shen, Z. A hierarchical MoP nanoflake array supported on Ni foam: a bifunctional electrocatalyst for overall water splitting. *Small Methods* **2**, 1700369 (2018).

87 Guo, Y., Fu, X., Zhang, B. & Peng, Z. Vertically standing MoP nanosheet arrays on Mo substrate: An integrated binder-free electrode for highly efficient and stable hydrogen evolution. J. Alloys Compd. **792**, 732-741 (2019).

88 Popczun, E. J. *et al.* Nanostructured nickel phosphide as an electrocatalyst for the hydrogen evolution reaction. *J. Am. Chem. Soc.* **135**, 9267-9270 (2013).

89 Liang, Y., Liu, Q., Asiri, A. M., Sun, X. & Luo, Y. Self-supported FeP nanorod arrays: a cost-effective 3D hydrogen evolution cathode with high catalytic activity. *ACS Catal.* **4**, 4065-4069 (2014).

90 Yan, X., Tian, L., He, M. & Chen, X. Three-dimensional crystalline/amorphous Co/Co3O4 core/shell nanosheets as efficient electrocatalysts for the hydrogen evolution reaction. *Nano lett.* **15**, 6015-6021 (2015).

91 Tian, J., Liu, Q., Asiri, A. M. & Sun, X. Self-supported nanoporous cobalt phosphide nanowire arrays: an efficient 3D hydrogen-evolving cathode over the wide range of pH 0–14. *J. Am. Chem. Soc.* **136**, 7587-7590 (2014).
